# Supplementary material for: Phage-assisted evolution of highly active cytosine base editors with enhanced selectivity and minimal sequence context preference
Source: Nat Commun. 2024 Feb 24;15:1697. doi: 10.1038/s41467-024-45969-7 (PMC10894238; doi:10.1038/s41467-024-45969-7)
Supplement: Supplementary file 1 — Supplementary Information [file 41467_2024_45969_MOESM1_ESM.pdf]

Supplementary Information

Phage-assisted evolution of highly active cytosine base editors with enhanced selectivity and minimal sequence context preference

Emily Zhang[1,2,3], Monica E. Neugebauer[1,2,3], Nicholas A. Krasnow[1,2,3], and David R. Liu[1,2,3*]

[1]Merkin Institute of Transformative Technologies in Healthcare, Broad Institute of MIT and Harvard, Cambridge, Massachusetts, USA

[2]Department of Chemistry and Chemical Biology, Harvard University, Cambridge, Massachusetts, USA

[3]Howard Hughes Medical Institute, Harvard University, Cambridge, Massachusetts, USA

* To whom correspondence should be addressed: drliu@fas.harvard.edu

Supplementary Figures

1. Evolved genotypes from PANCE and PACE.
2. Comparison of TadDE N46I and TadDE N46T variants from PANCE.
3. Energy calculation for TadA* stability with and without Y73P.
4. *E. coli* profiling of TadDE-evolved CBEs at every 5' and 3' sequence context at position 6 in the protospacer (RBS=SD8).
5. *E. coli* profiling of TadDE-evolved CBEs at every 5' and 3' sequence context at position 6 in the protospacer (RBS=sd5).
6. Sequence context motifs for a target cytidine at every 5' and 3' sequence context at positions 1-14 in the protospacer.
7. Sequence context motifs for a target adenine at every 5' and 3' sequence context at positions 1-14 in the protospacer.
8. *E. coli* profiling of TadDE-evolved CBEs for cytidine deamination at every 5' and 3' sequence context at positions 1-10 in the protospacer.
9. *E. coli* profiling of TadDE-evolved CBEs for adenine deamination at every 5' and 3' sequence context at positions 1-10 in the protospacer.
10. Reversion analysis of TadDE-evolved CBE variants.
11. Addition of mutations to TadCBEd.
12. Full comparison of base editors at four SpCas9 target sites.
13. Full comparison of base editors at four eNme2-C Cas9 target sites.
14. Indels by SpCas9 variants at four SpCas9 target sites.
15. Indels by eNme2-C Cas9 variants at four eNme2-C Cas9 target sites.
16. On-target and Cas-dependent editing of known off-target sites for *HEK3*.
17. On-target and Cas-dependent editing of known off-target sites for *HEK4*.
18. On-target and Cas-dependent editing of known off-target sites for *EMX1*.
19. On-target and Cas-dependent editing of known off-target sites for *BCL11a*.
20. On-target editing at *EMX1* correlated to Cas-independent off-target editing.
21. Cas-independent off-target editing at six genomic SaCas9 R-loops.
22. On-target editing at *EMX1* correlated to RNA off-target editing.
23. Comparison of peak editing of base editors at four SpCas9 target sites with statistical significance.
24. Comparison of peak editing of base editors at four eNme2C-Cas9 target sites with statistical significance.

25. Peak editing of base editors at 3 *PCSK9* target sites with statistical significance.
26. Cas-independent off-target editing at six genomic SaCas9 R-loops with statistical significance.
27. Cas-dependent off-target editing of peak editing of known off-target sites for *HEK3* with statistical significance.
28. Cas-dependent off-target editing of peak editing of known off-target sites for *HEK4* with statistical significance.
29. Cas-dependent off-target editing of peak editing of known off-target sites for *EMX1* with statistical significance.
30. Cas-dependent off-target editing of peak editing of known off-target sites for *BCL11a* with statistical significance.
31. RNA off-target editing with statistical significance.

Supplementary Tables

1. Selectivity of TadDE-evolved CBEs calculated from *E. coli* profiling library (SD8, position 6)
2. Selectivity of TadDE-evolved CBEs calculated from *E. coli* profiling library (sd2, positions 4-8)
3. Plasmids and selection phage (SP) used in this work.
4. Promoter and RBS sequences for plasmids and phage used in evolution.
5. cDNA amplicon sequences and primers for RNA off-target analysis.
6. Primers for generating base editor amplicons for IVT.
7. Chemically synthesized guide RNAs used for fibroblast experiments.

Supplementary Notes

1. Evolved deaminase amino acid sequences.
2. Sequences of Cas9 domains.

a

TadA-8e	R26	V28	N46	A48	Q71	Y73	H96	Q154
TadDE	G	A		R		S	N	H
L1-1	G	A	I	R		P	N	
L1-2	G	A	I	R		P	N	
L1-3	G	A	T	R		S	N	H
L1-4	G	A		R		S	N	H
L2-1	G	A	I	R		S	N	
L2-2	G	A	T	R		S	N	
L2-3	G	A		R	S	S	N	

b

TadA-8e	R26	V28	N46	A48	Y73	T79	H96	G105
TadDE	G	A		R	S		N	
L1-1	G	A	L	R	P			
L1-2	G	A	L	R	P		N	
L1-3	G	A			P		N	
L1-4	G	A	V	R	S		N	
L2-1	G	A	V	R	P		N	
L2-2	G		L					
L2-3	G	A	I	R	P		N	
L2-4	G	A	V	R	P		N	S
L3-1	G	A		P	H	P	N	
L3-2	G		I				N	

c

TadA-8e	R26	V28	N46	A48	Q71	Y73	H96	A162
TadDE	G	A		R		S	N	
72 h	G	A	V	R		P	N	
72 h	G	A	C	R		P	N	V
72 h	G	A	L	R		S	N	
72 h	G	A	V	R		P	N	
94 h	G	A	C	R		P	N	V
94 h	G	A	C	R		P	N	V
94 h	G	A	C	R			N	V
94 h	G	A	V	R	H	P	N	
118 h	G	A	C	R		P	N	
118 h	G	A	C	R		P	N	
118 h	G	A	C	R		P	N	
118 h	G	A	C	R		P	N	
118 h	G	A	C	R		P	N	V
118 h	G	A	C	R		P	N	

d

TadA-8e	R26	V28	L34	N46	A48	R64	Y73	H96
TadDE	G	A			R		S	N
72 h	G	A		V	R		P	N
72 h	G	A	M	L	R		P	N
72 h	G	A		C	R		P	N
72 h	G	A		V	R		S	N
94 h	G	A		V	P		S	N
94 h	G	A		L	R		P	N
94 h	G	A		L	P	K	P	N

Supplementary Figure 1. Evolved genotypes from PANCE and PACE. (a) Genotypes from PANCE lagoons (L1–L2) after 6 passages of PANCE. **(b)** Genotypes from PANCE lagoons (L1–L3) after 5 passages of PANCE using an NNK library at position N46. **(c)** Genotypes from PACE lagoon (L1) after PANCE using an NNK library at position N46. Phage were sequenced at the time points as indicated in the table. **(d)** Genotypes at various timepoints from PACE lagoon (L2) after PANCE using an NNK library at position N46.

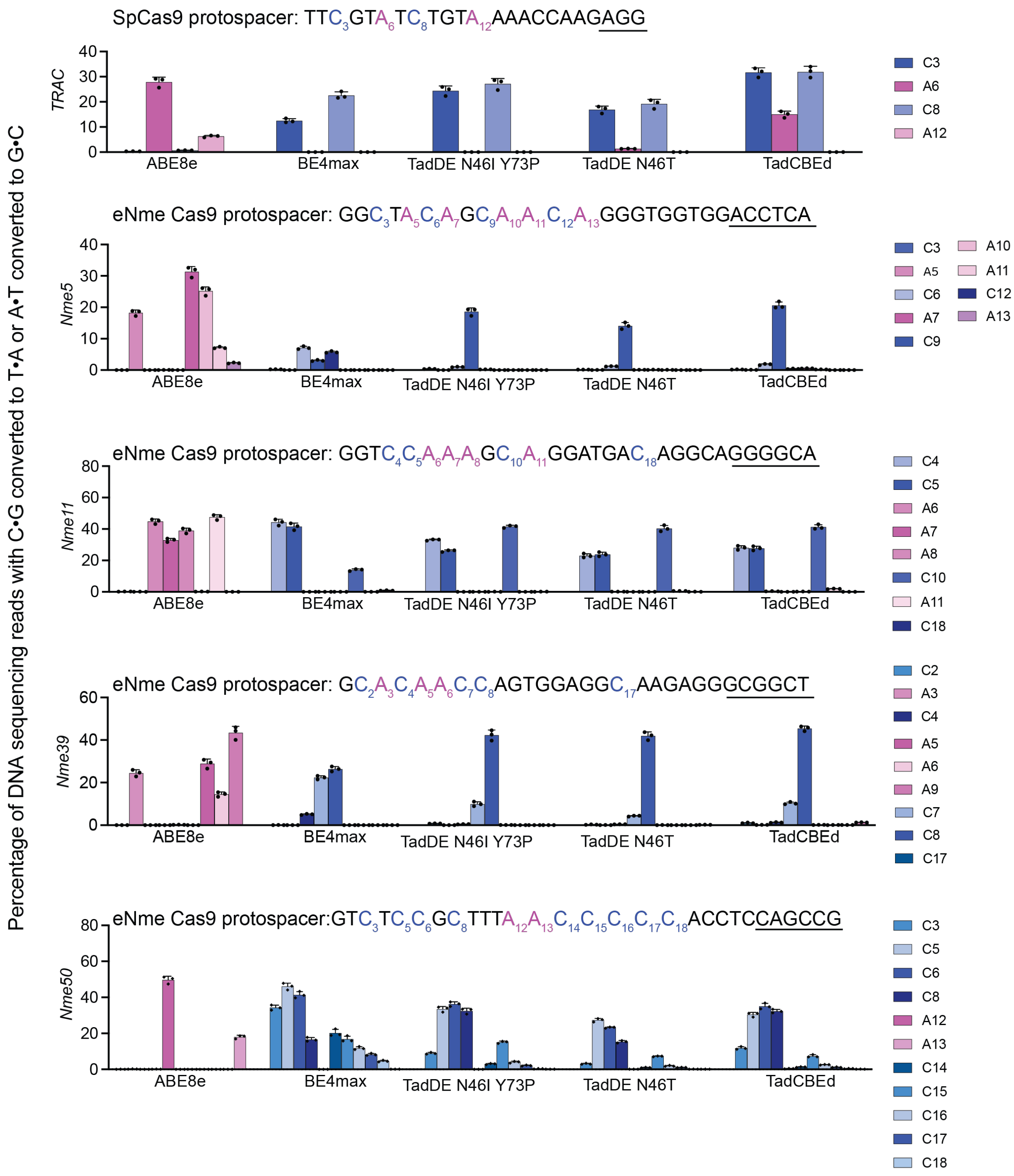

Supplementary Figure 2. Comparison of TadDE N46I and TadDE N46T variants from PANCE. The specified base editors using SpCas9 or eNmeC-Cas9 nickase domains in the BE4max architecture were transfected into HEK293T cells with a guide RNA targeting one of five genomic loci as shown in each graph. The mutations in the newly evolved variants are listed relative to TadDE. Target cytosines are blue, target adenines are magenta, and PAM sequences are underlined. C•G-to-T•A base editing is shown in shades of blue, and A•T-to-G•C base editing is shown in magenta. Dots represent individual values and bar values represent mean±s.d. of n=3 independent biological replicates. Source data are provided as a Source Data file.

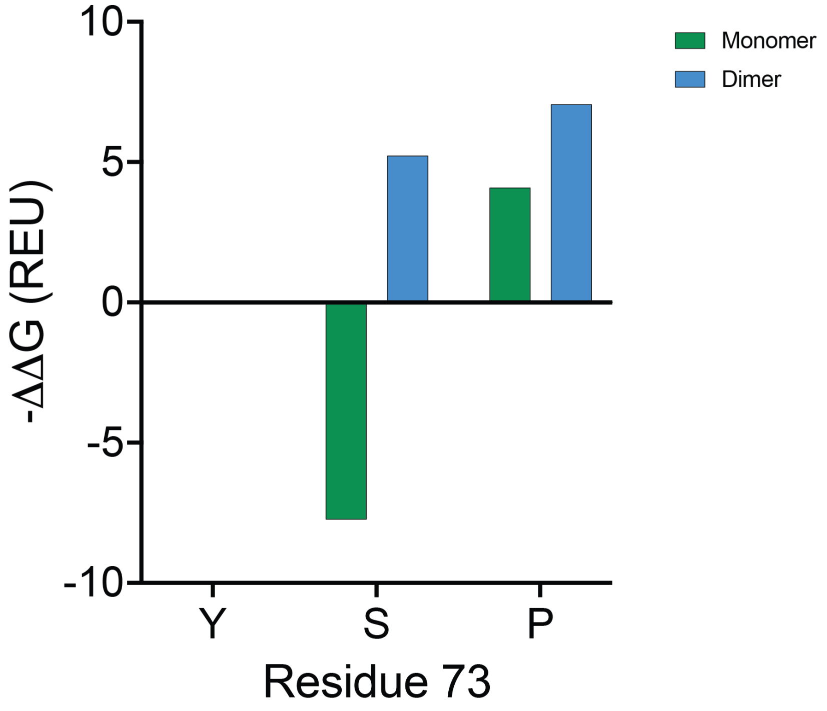

Supplementary Figure 3. Energy calculation for TadA* stability with and without Y73P. Rosetta was used to calculate the energy of the TadA* monomer and dimer with and without Y73P. ΔΔG represents predicts how a single point mutation is predicted to affect stability. Source data are provided as a Source Data file.

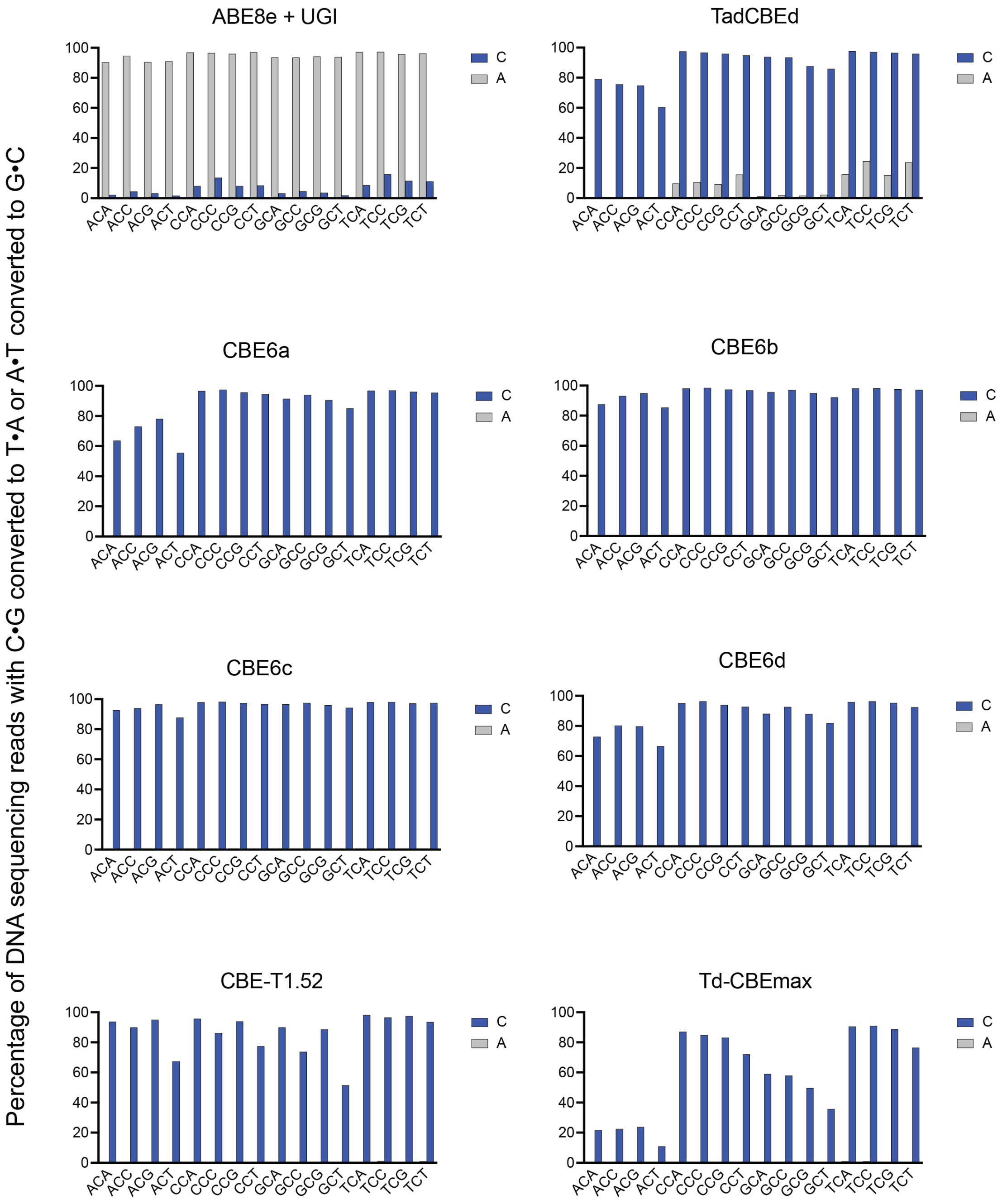

Supplementary Figure 4. *E. coli* profiling of TadDE-evolved CBEs at every 5' and 3' sequence context at position 6 in the protospacer (RBS=SD8). *E. coli* 10-Beta cells were transformed with specified base editors using dSpCas9 domains in the BE4max architecture or ABE8e and made competent for electroporation. A 32-member library with the target base at position 6 of the protospacer was electroporated into the *E. coli*. Each library member contains a matched protospacer and guide pair. Base editor expression was induced by addition of arabinose (RBS=SD8), and cells were grown overnight at 37 °C. Cells were harvested, and the target plasmid was analyzed by HTS. C•G-to-T•A base editing is shown in blue, and A•T-to-G•C editing is shown in grey. Each bar value is correlated to one of the dots presented in Figure 2b. Source data are provided as a Source Data file.

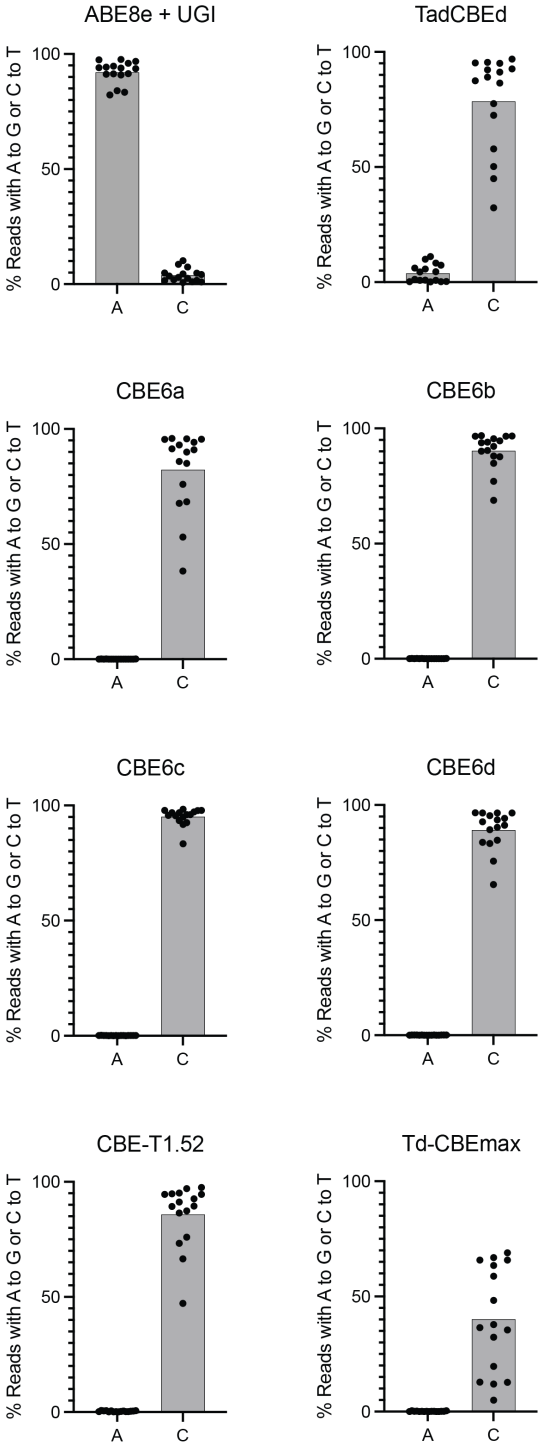

Supplementary Figure 5. *E. coli* profiling of TadDE-evolved CBEs at every 5' and 3' sequence context at position 6 in the protospacer (RBS=sd5). Base editing in *E. coli* using the 32-member library at position 6 of the protospacer (RBS=sd5). C•G-to-T•A edits and A•T-to-G•C edits are shown separately as bars. Dots represent the mean of n=3 independent biological replicates for each 5' and 3' sequence context. Source data are provided as a Source Data file.

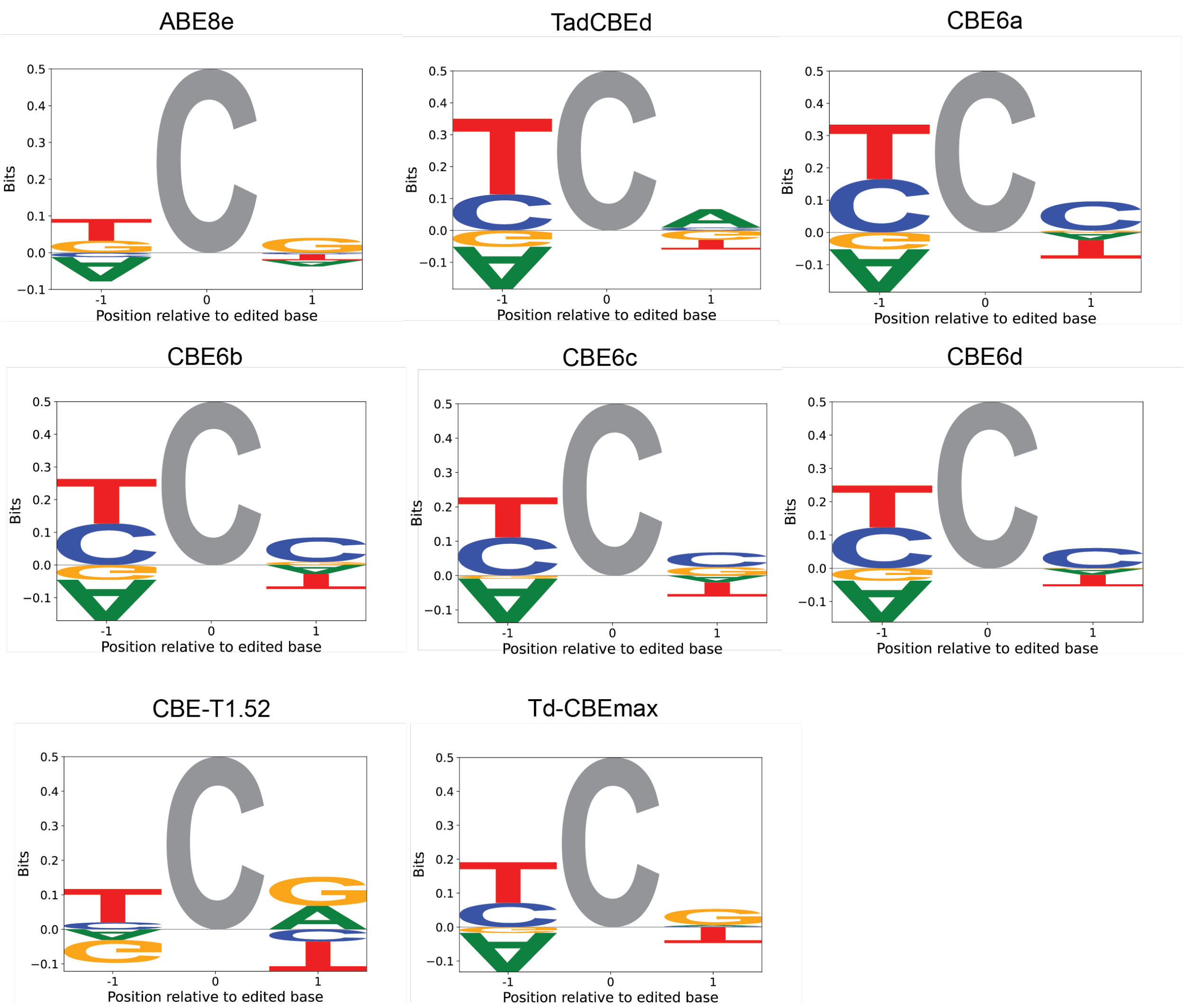

Supplementary Figure 6. Sequence context motifs for a target cytidine at every 5' and 3' sequence context at positions 1-14 in the protospacer. Sequence motifs (pos. 1-14) of CBE variants and ABE8e (RBS=sd2) when tested on a library of substrates designed to contain the target base (C) at protospacer positions 1-14 flanked by variable nucleotides (n=2 independent biological replicates). Source data are provided as a Source Data file.

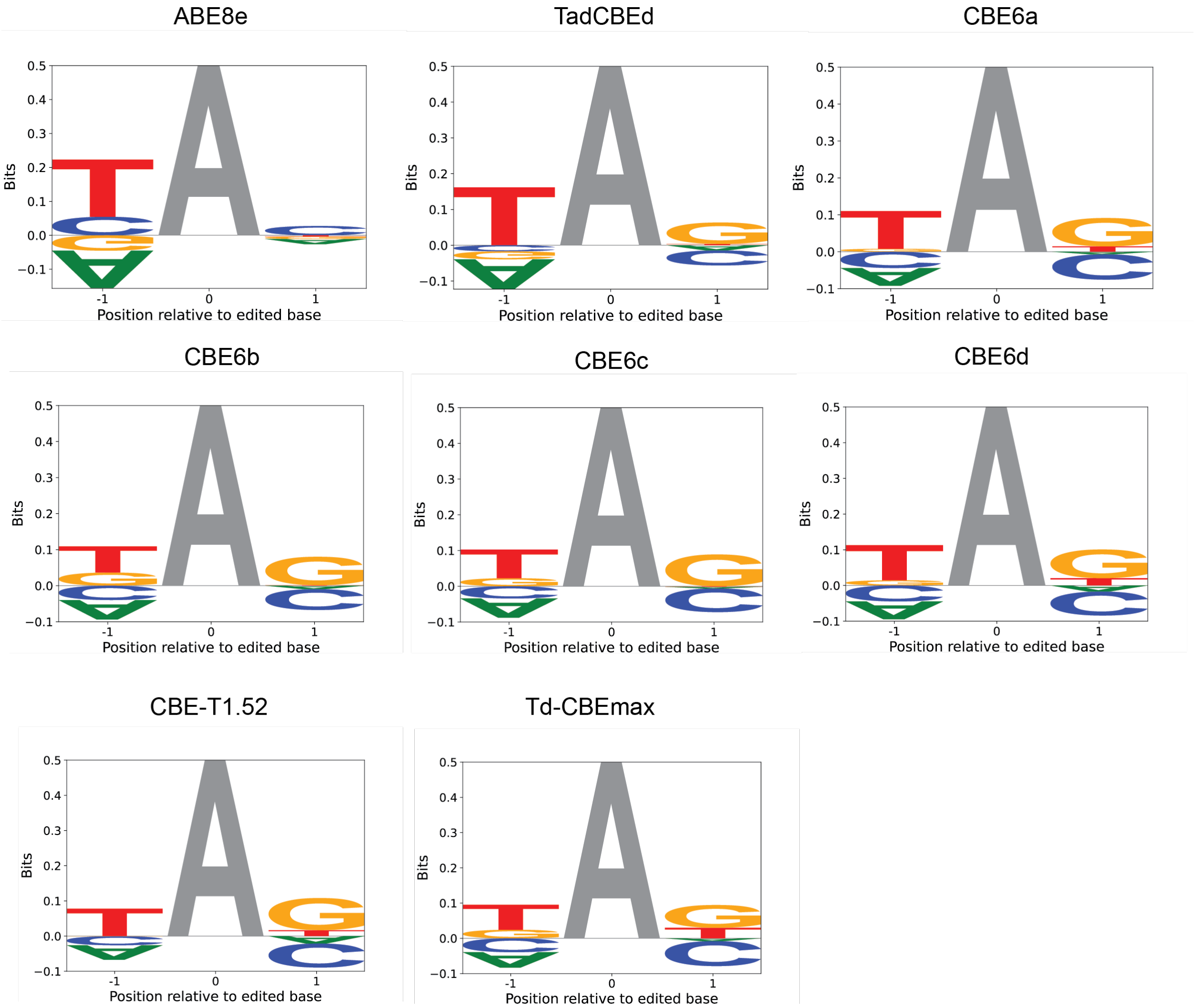

Supplementary Figure 7. Sequence context motifs for a target adenine at every 5' and 3' sequence context at positions 1-14 in the protospacer. Sequence motifs (pos. 1-14) of CBE variants and ABE8e (RBS=sd2) when tested on a library of substrates designed to contain the target base (A) at protospacer positions 1-14 flanked by variable nucleotides (n=2 independent biological replicates). Source data are provided as a Source Data file.

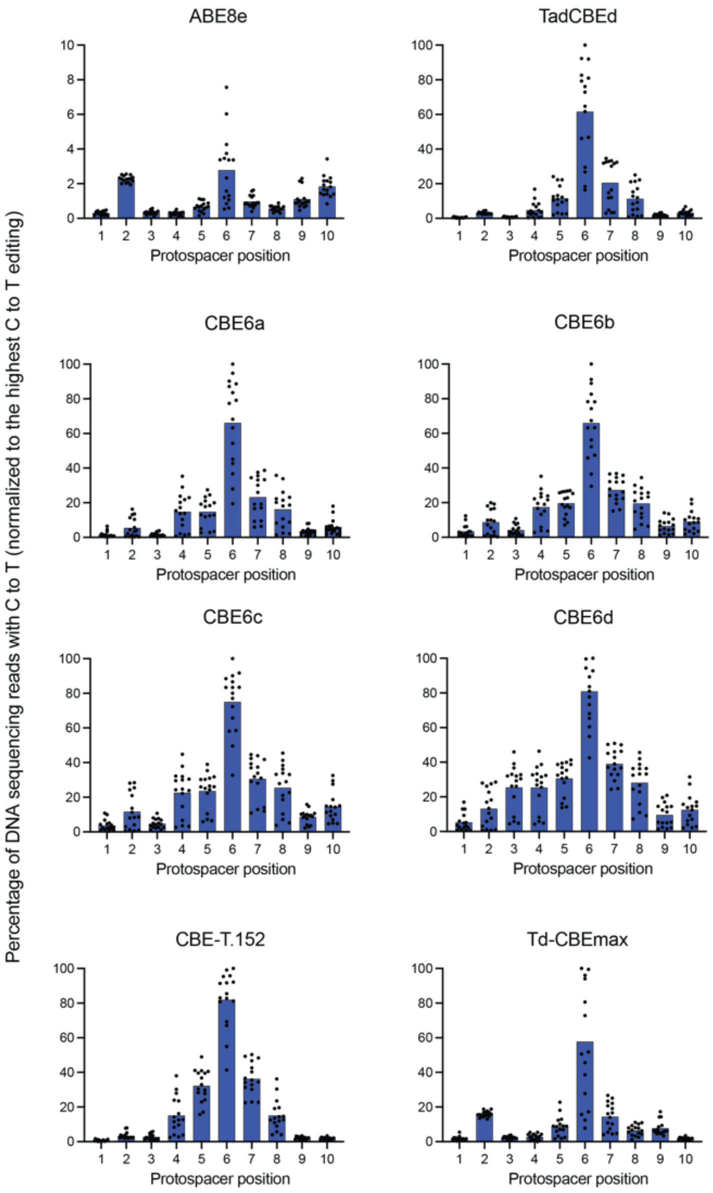

Supplementary Figure 8. ***E. coli*** **profiling of TadDE-evolved CBEs for cytidine deamination at every 5' and 3' sequence context at positions 1-10 in the protospacer.** *E. coli* 10-Beta cells were transformed with specified base editors using dSpCas9 domains in the BE4max architecture or ABE8e and made competent for electroporation. A 448-member library with the target base at positions ranging from 1-14 of the protospacer was electroporated into the *E. coli*. Each library member contains a matched protospacer and guide pair. Base editor expression was induced by addition of arabinose (RBS=sd2), and cells were grown overnight at 37 °C. Cells were harvested, and the target plasmid was analyzed by HTS. C•G-to-T•A base editing is shown in blue. Each dot represents the mean percentage of sequencing reads containing the specified edit for a given sequence context (n=2 independent biological replicates). Data were normalized to the highest C•G-to-T•A activity for CBE variants or A•T-to-G•C activity for ABE8e. Source data are provided as a Source Data file.

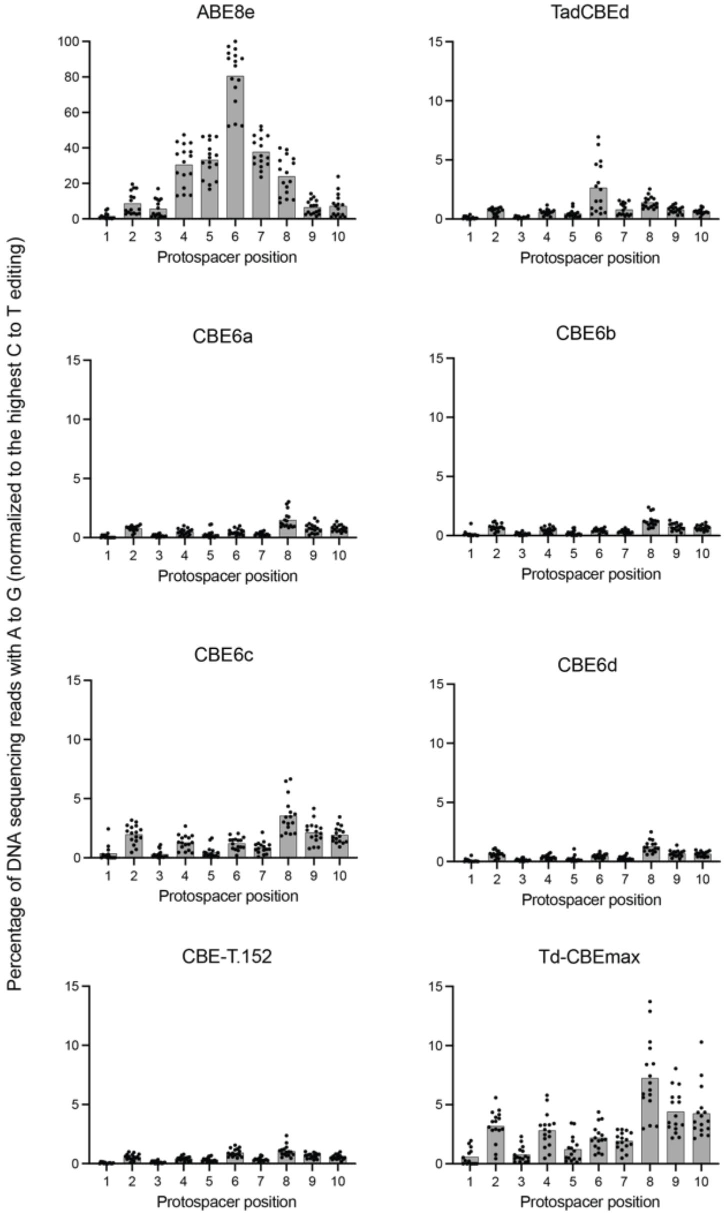

Supplementary Figure 9. *E. coli* profiling of TadDE-evolved CBEs for adenine deamination at every 5' and 3' sequence context at positions 1-10 in the protospacer. *E. coli* 10-Beta cells were transformed with specified base editors using dSpCas9 domains in the BE4max architecture or ABE8e and made competent for electroporation. A 448-member library with the target base at positions ranging from 1-14 of the protospacer was electroporated into the *E. coli*. Each library member contains a matched protospacer and guide pair. Base editor expression was induced by addition of arabinose (RBS=sd2), and cells were grown overnight at 37 °C. Cells were harvested, and the target plasmid was analyzed by HTS. A•T-to-G•C base editing is shown in gray. Each dot represents the mean percentage of sequencing reads containing the specified edit for a given sequence context (n=2 independent biological replicates). Data were normalized to the highest C•G-to-T•A activity for CBE variants or A•T-to-G•C activity for ABE8e. Source data are provided as a Source Data file.

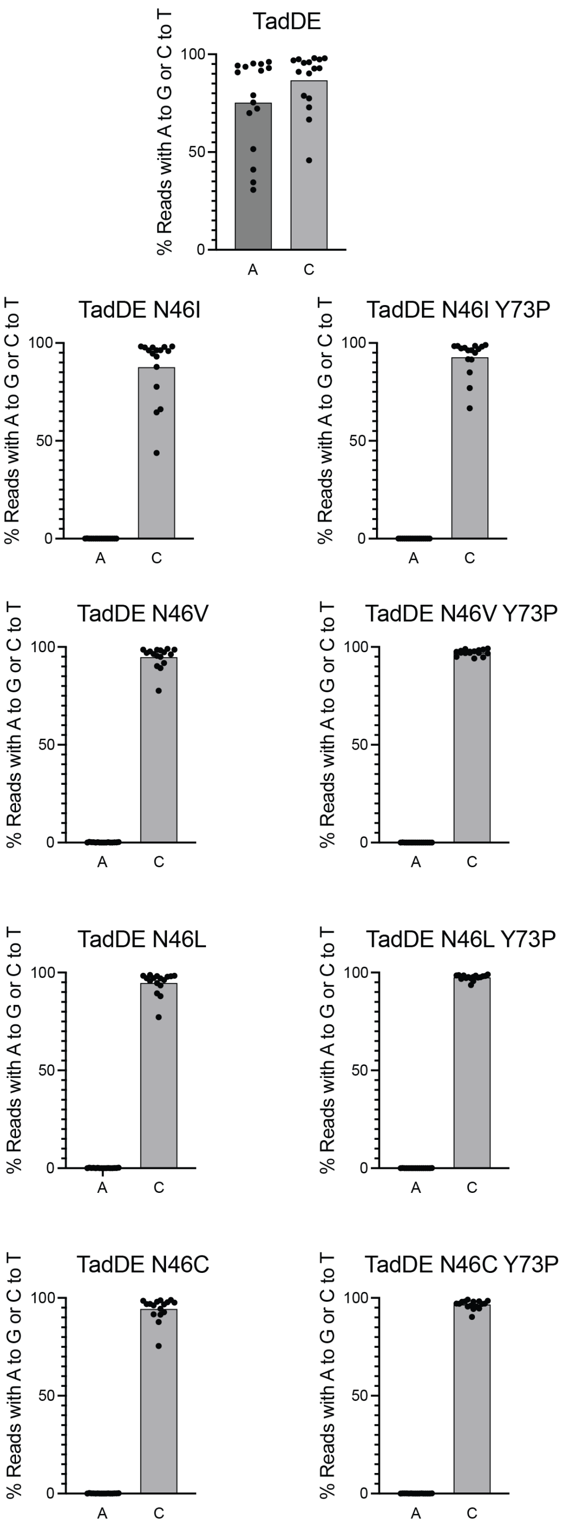

Supplementary Figure 10. Reversion analysis of TadDE-evolved CBE variants. Base editing in *E. coli* using the 32-member library at position 6 of the protospacer (RBS=SD8). Mutations shown are relative to TadDE. C•G-to-T•A edits and A•T-to-G•C edits are shown separately as bars. Dots represent the mean of n=3 independent biological replicates for each 5' and 3' sequence context. Source data are provided as a Source Data file.

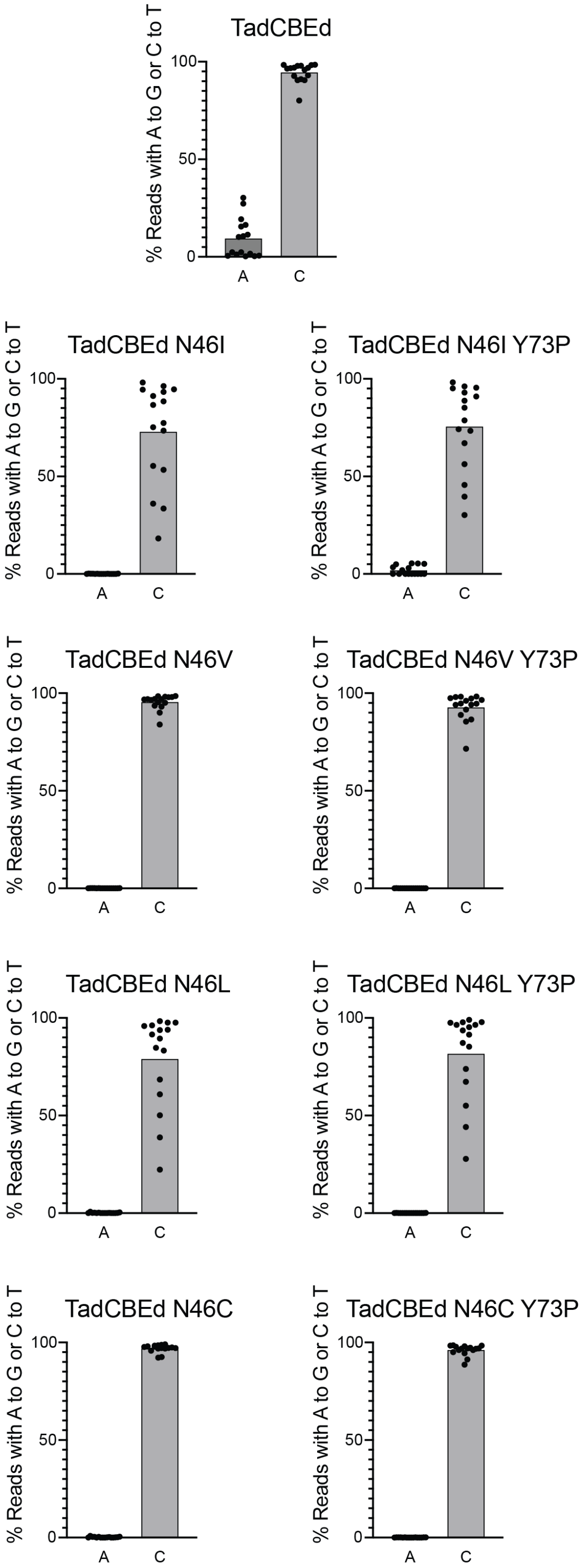

Supplementary Figure 11. Addition of mutations to TadCBEd. Base editing in *E. coli* using the 32-member library at position 6 of the protospacer (RBS=SD8). Mutations shown are relative to TadCBEd. C•G-to-T•A edits and A•T-to-G•C edits are shown separately as bars. Dots represent the mean of n=3 independent biological replicates for each 5' and 3' sequence context. Source data are provided as a Source Data file.

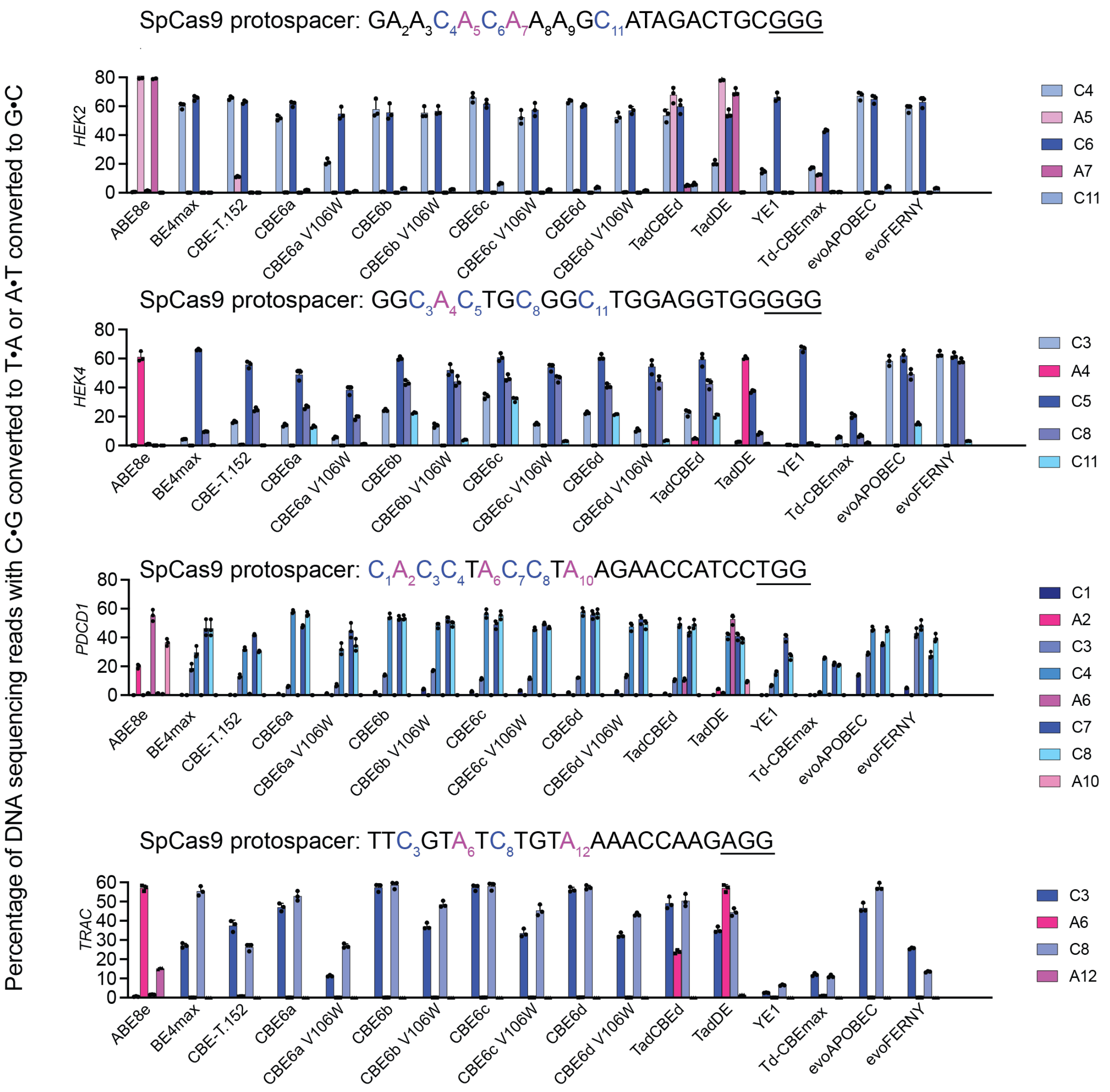

Supplementary Figure 12. Full comparison of base editors at four SpCas9 target sites. The specified base editors using SpCas9 nickase domains in the BE4max architecture were transfected into HEK293T cells with a guide RNA targeting one of four genomic loci as shown in each graph. HEK293T site 2 is abbreviated *HEK2*, and HEK293T site 4 is abbreviated *HEK4*. Target cytosines are blue, target adenines are magenta, and PAM sequences are underlined. C•G-to-T•A base editing is shown in shades of blue, and A•T-to-G•C base editing is shown in magenta. Dots represent individual values and bar values represent mean±s.d. of n=3 independent biological replicates. Source data are provided as a Source Data file.

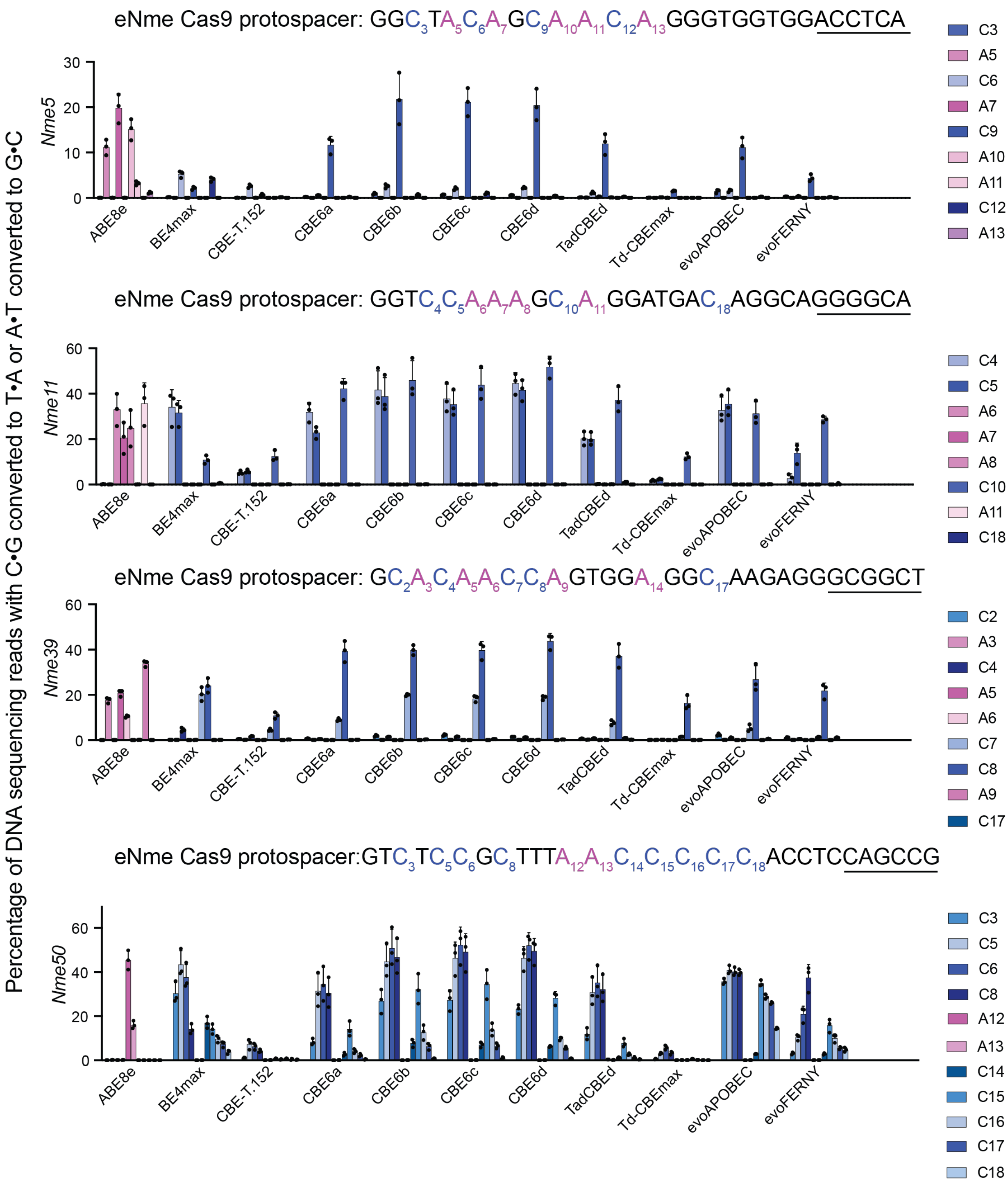

Supplementary Figure 13. Full comparison of base editors at four eNmeC-Cas9 target sites. The specified base editors using eNmeC-Cas9 nickases in the BE4max architecture were transfected into HEK293T cells with a guide RNA targeting the protospacer shown in each graph. Target cytosines are blue, target adenines are magenta, and PAM sequences are underlined. C•G-to-T•A base editing is shown in shades of blue, and A•T-to-G•C base editing is shown in magenta. Dots represent individual values and bar values represent mean±s.d. of n=3 independent biological replicates. Source data are provided as a Source Data file.

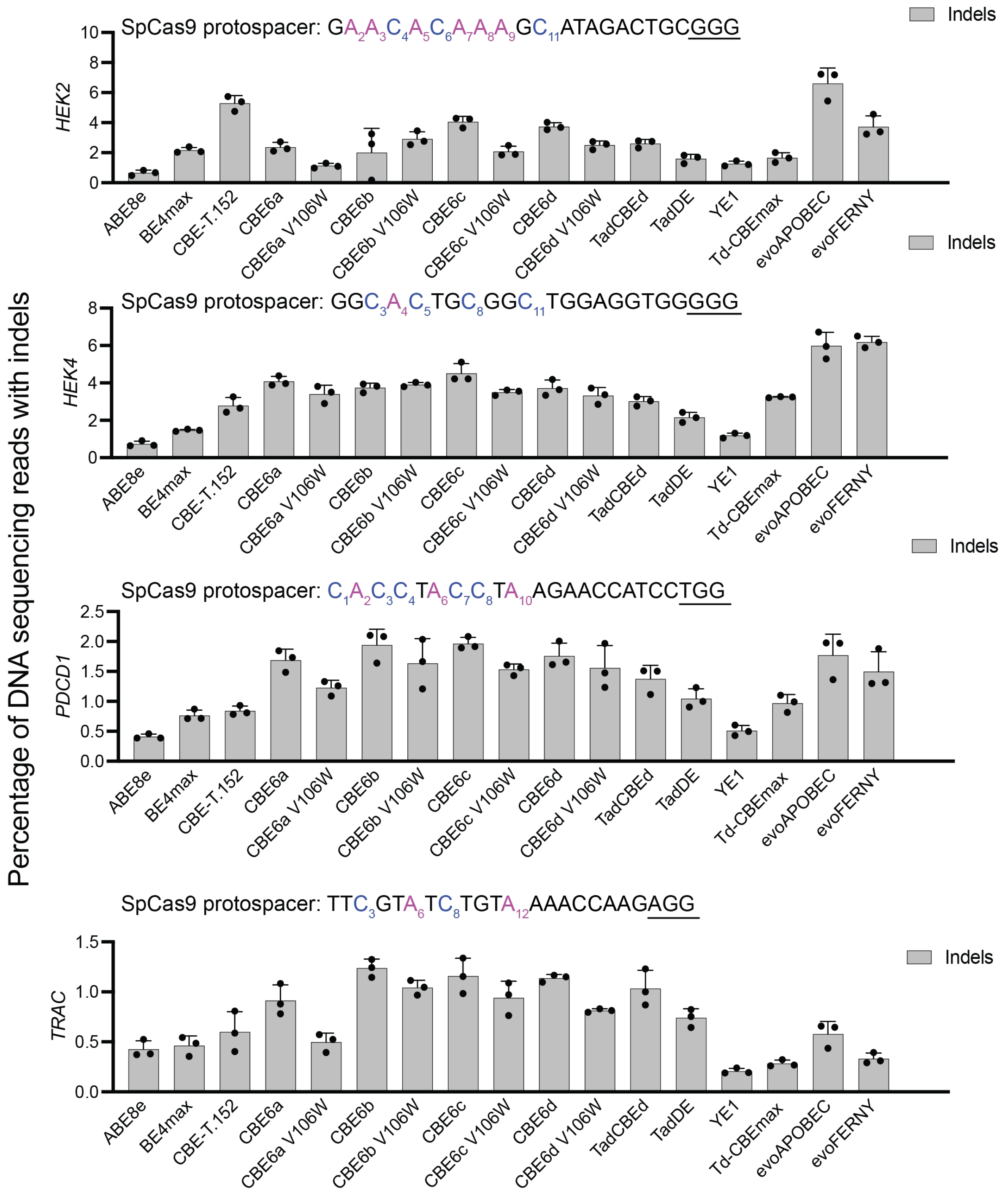

Supplementary Figure 14. Indels by SpCas9 variants at four SpCas9 target sites. The specified base editors using SpCas9 nickase domains in the BE4max architecture were transfected into HEK293T cells with a guide RNA targeting one of four genomic loci as shown in each graph. Target cytosines are blue, target adenines are magenta, and PAM sequences are underlined. Indels are shown in grey. Dots represent individual values and bar values represent mean±s.d. of n=3 independent biological replicates. The data here are indels from the editing data presented in Supplementary Fig. 12. HEK293T site 2 is abbreviated *HEK2*, and HEK293T site 4 is abbreviated *HEK4*. Source data are provided as a Source Data file.

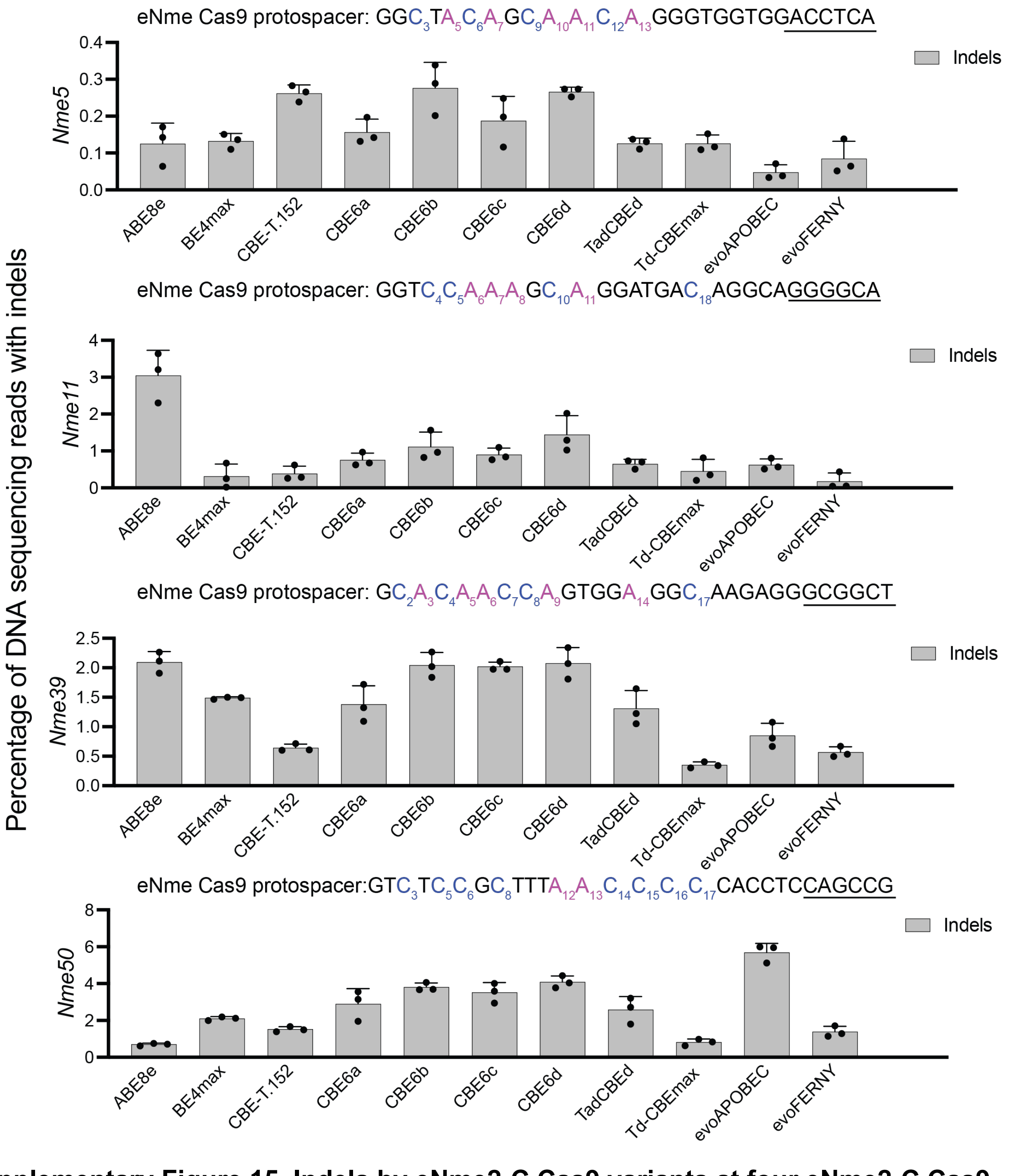

Supplementary Figure 15. Indels by eNme2-C Cas9 variants at four eNme2-C Cas9 target sites. The specified base editors using eNme2-C Cas9 nickase domains in the BE4max architecture were transfected into HEK293T cells with a guide RNA targeting one of four protospacers as shown in each graph. Target cytosines are blue, target adenines are magenta, and PAM sequences are underlined. Indels are shown in grey. Dots represent individual values and bar values represent mean±s.d. of n=3 independent biological replicates. The data here are indels from the editing data presented in Supplementary Fig. 13. Source data are provided as a Source Data file.

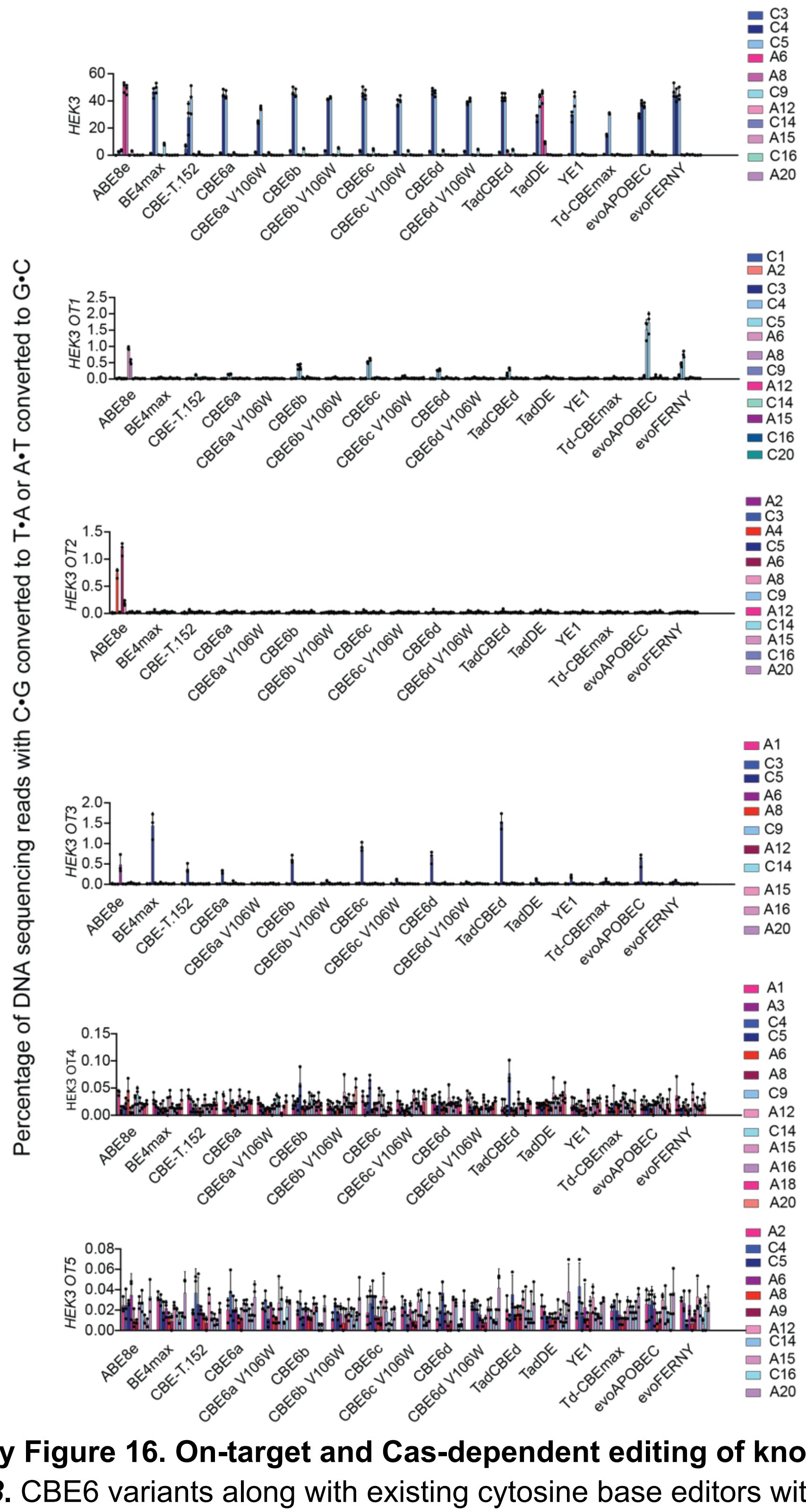

Supplementary Figure 16. On-target and Cas-dependent editing of known off-target sites for *HEK3*. CBE6 variants along with existing cytosine base editors with SpCas9 nickases in the BE4max architecture were transfected into HEK293T cells with a guide RNA targeting *HEK3*. HEK293T site 3 is abbreviated *HEK3*. Dots represent individual values and bar values represent mean±s.d. of n=3 independent biological replicates. Source data are provided as a Source Data file.

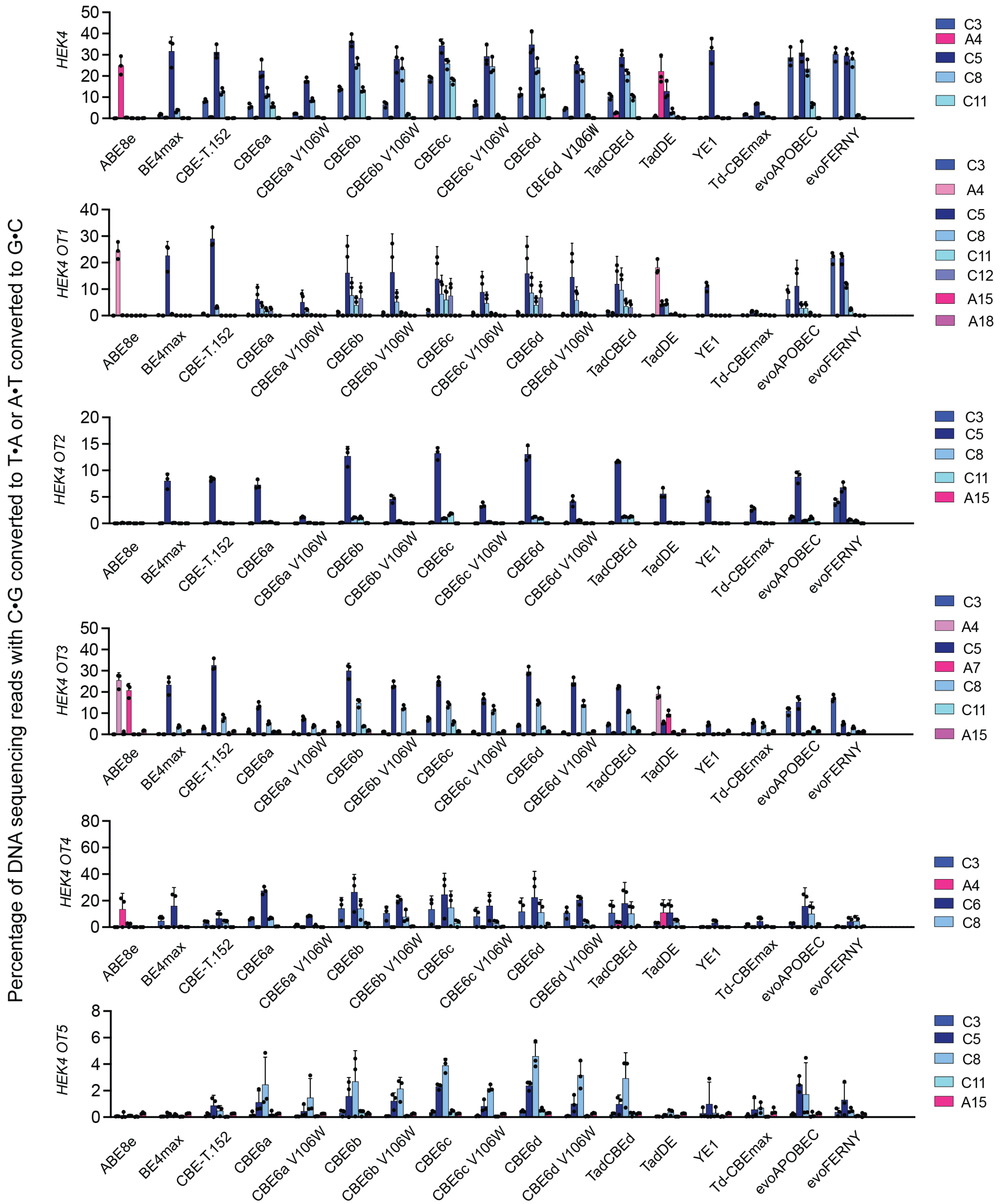

Supplementary Figure 17. On-target and Cas-dependent editing of known off-target sites for *HEK4*. CBE6 variants along with existing cytosine base editors with SpCas9 nickases in the BE4max architecture were transfected into HEK293T cells with a guide RNA targeting *HEK4*. HEK293T site 4 is abbreviated *HEK4*. Dots represent individual values and bar values represent mean±s.d. of n=3 independent biological replicates. Source data are provided as a Source Data file.

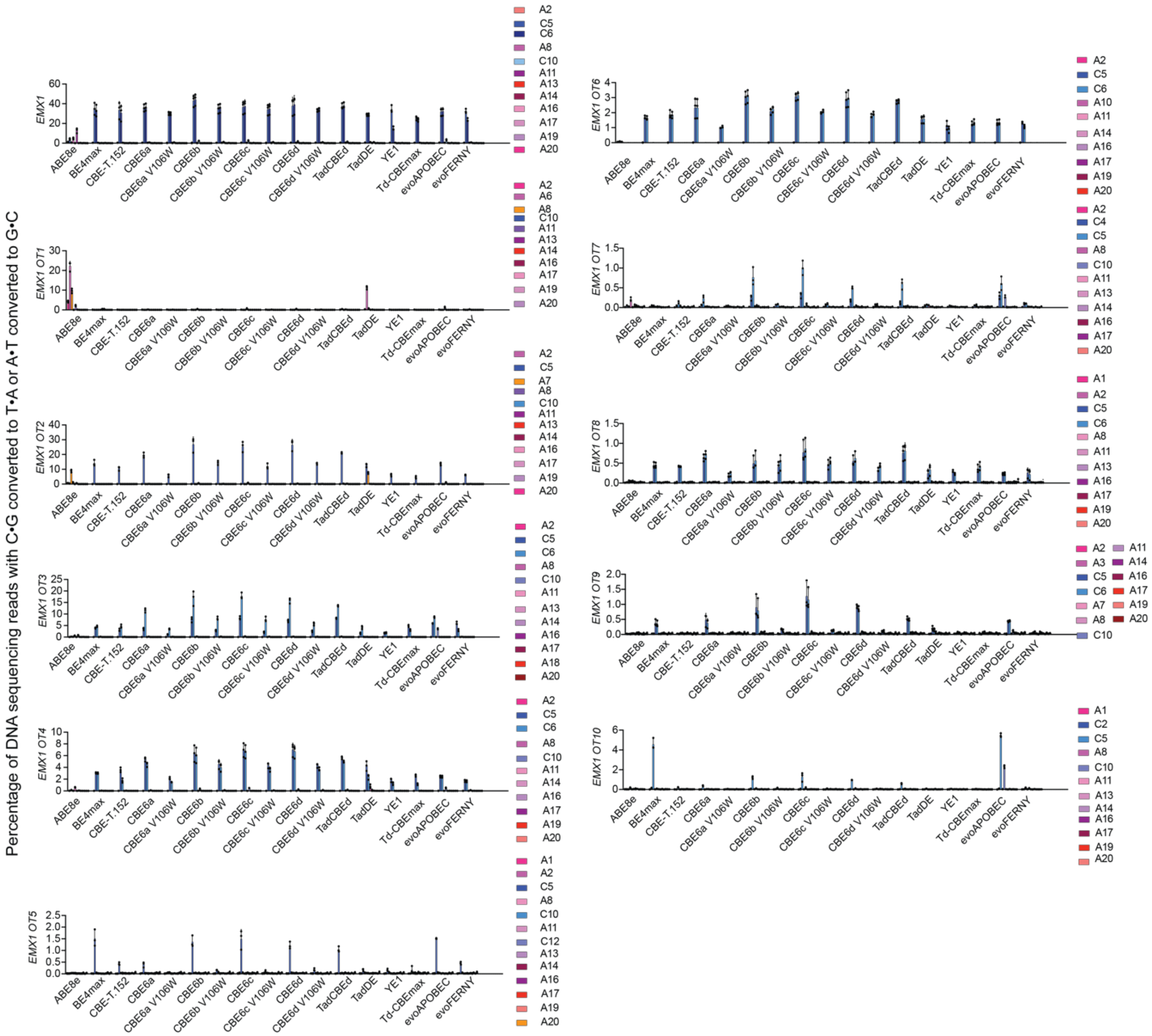

Supplementary Figure 18. On-target and Cas-dependent editing of known off-target sites for *EMX1*. CBE6 variants along with existing cytosine base editors with SpCas9 nickases in the BE4max architecture were transfected into HEK293T cells with a guide RNA targeting *EMX1*. Dots represent individual values and bar values represent mean±s.d. of n=3 independent biological replicates. Source data are provided as a Source Data file.

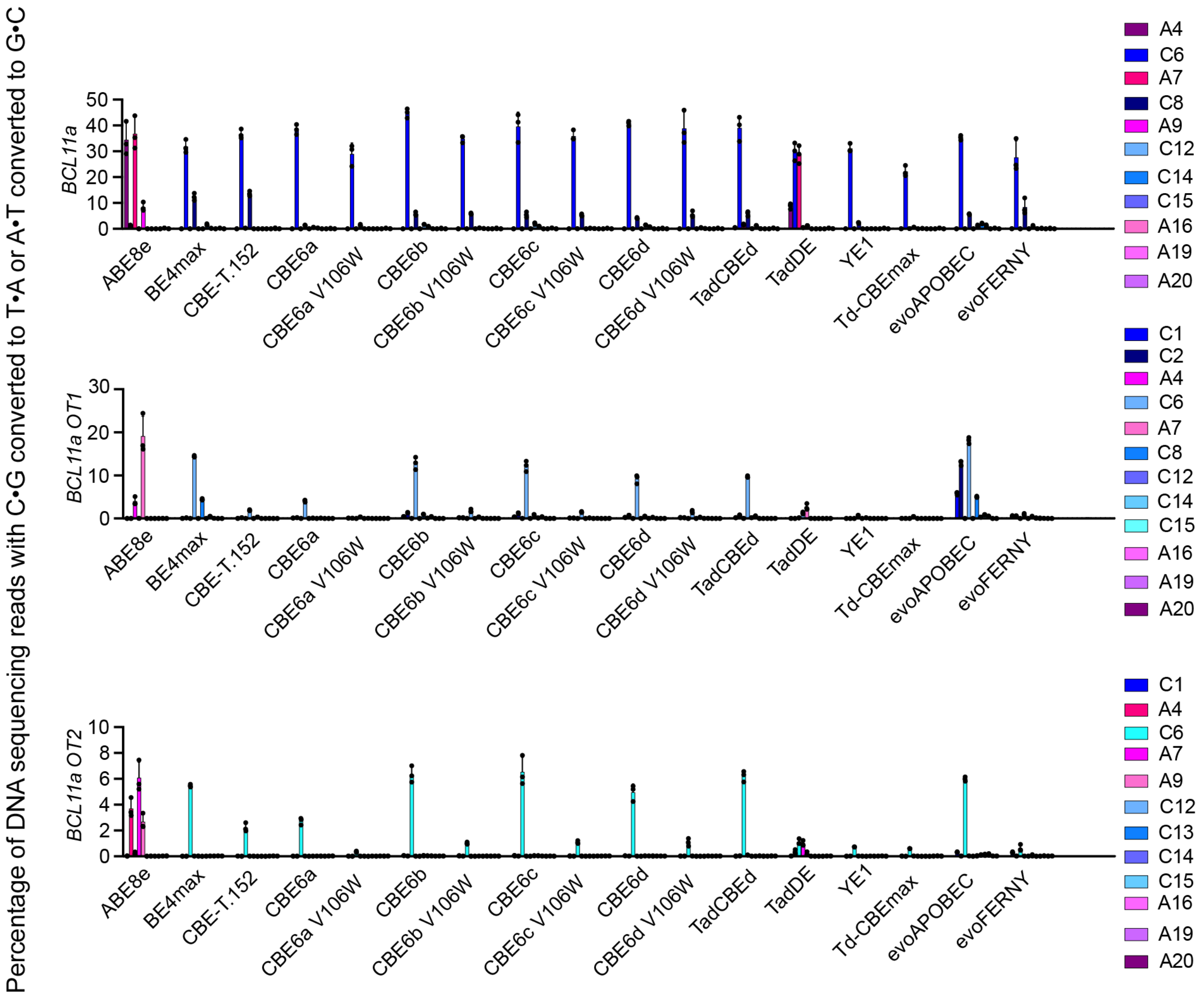

Supplementary Figure 19. On-target and Cas-dependent editing of known off-target sites for *BCL11a*. CBE6 variants along with existing cytosine base editors with SpCas9 nickases in the BE4max architecture were transfected into HEK293T cells with a guide RNA targeting *BCL11a*. Dots represent individual values and bar values represent mean±s.d. of n=3 independent biological replicates. Source data are provided as a Source Data file.

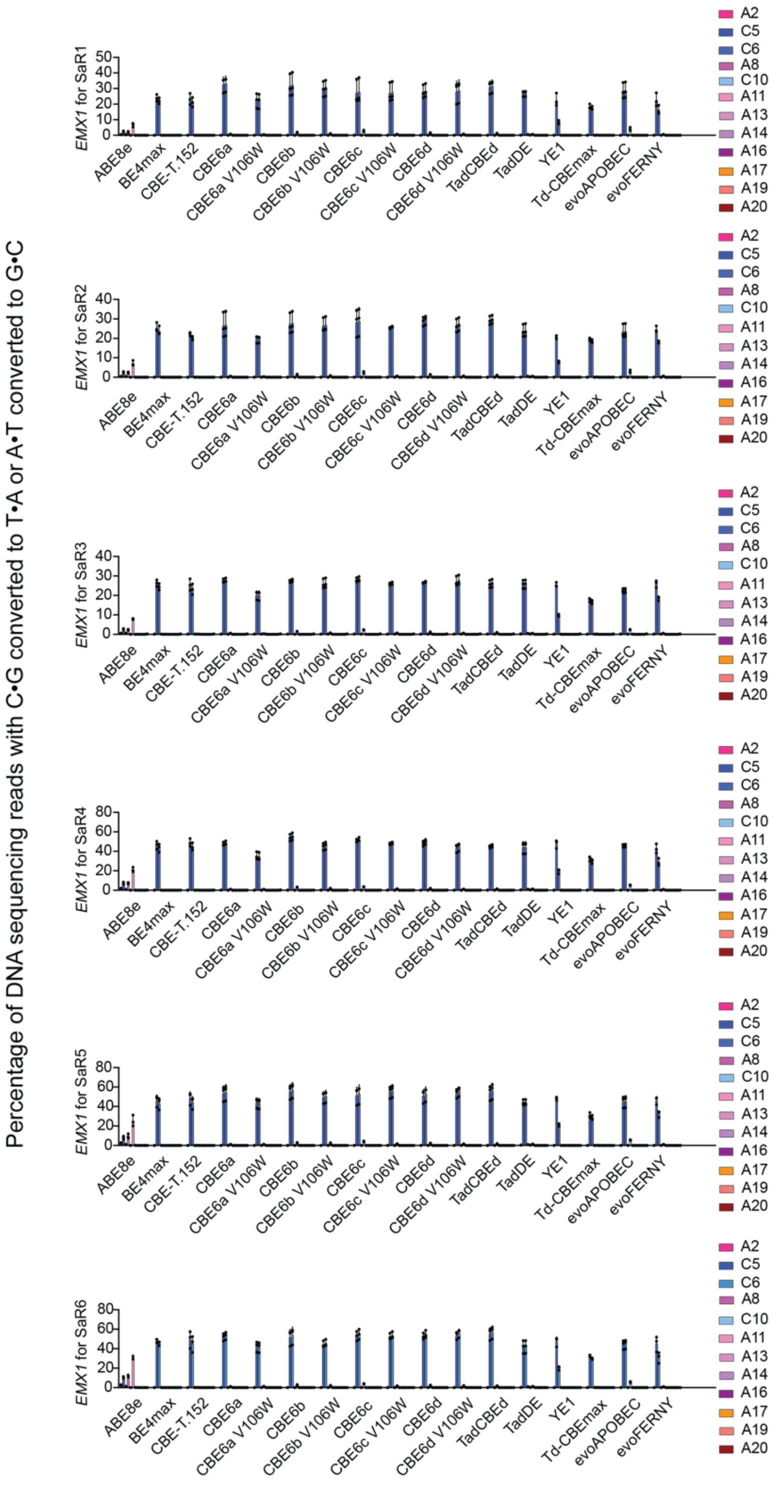

Supplementary Figure 20. On-target editing at *EMX1* correlated to Cas-independent off-target editing. CBE6 variants along with existing cytosine base editors with SpCas9 nickases in the BE4max architecture were transfected into HEK293T cells with an SpCas9 guide RNA targeting *EMX1* along with an SaCas9 guide RNA. Dots represent individual values and bar values represent mean±s.d. of n=3 independent biological replicates. Source data are provided as a Source Data file.

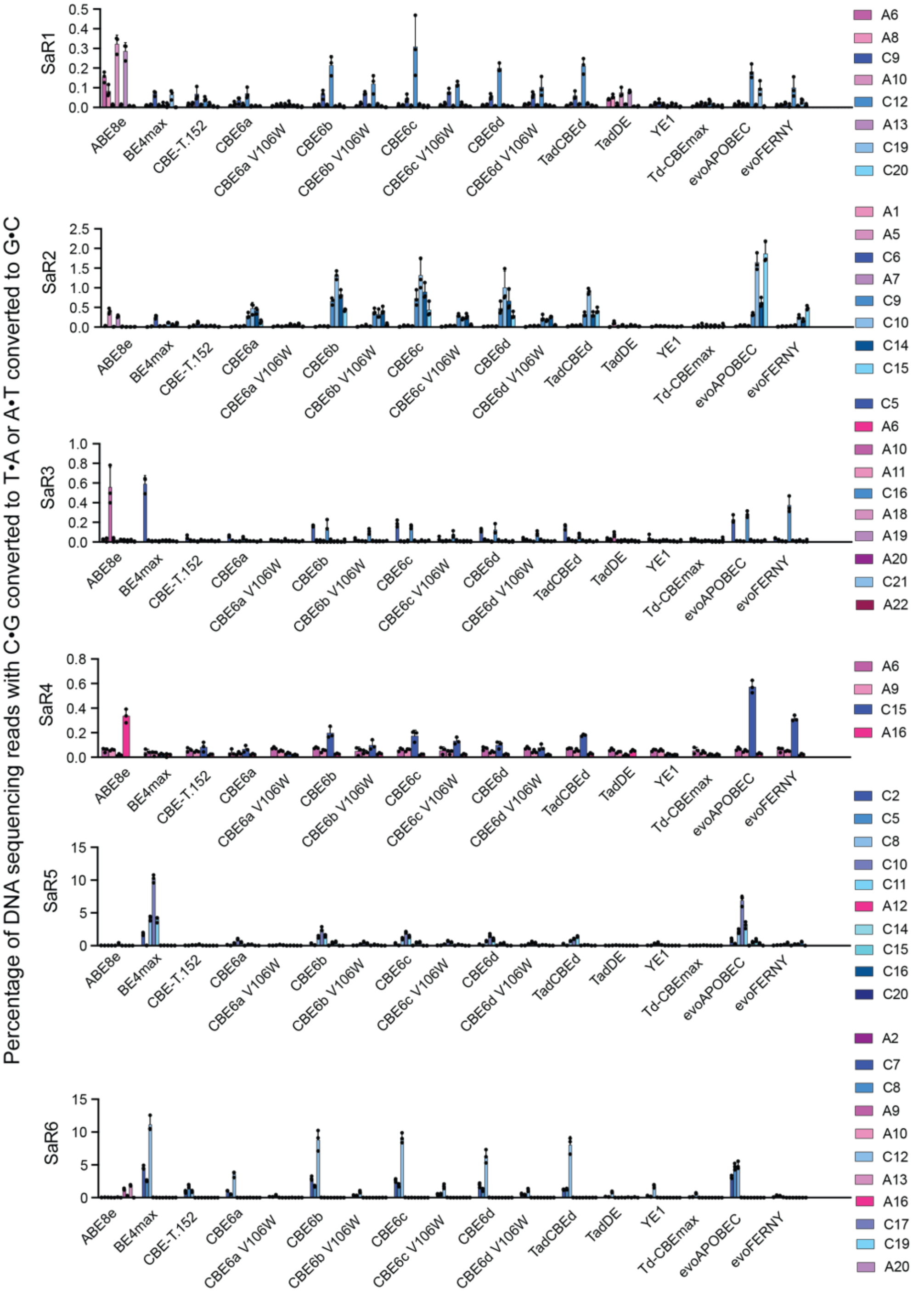

Supplementary Figure 21. Cas-independent off-target editing at six genomic SaCas9 R-loops. The orthogonal R-loop assay was performed on CBE variants in the BE4max architecture. HEK293T cells were transfected with the base editor and an SpCas9 sgRNA targeting *EMX1* as the on-target locus. Simultaneously, orthogonal dSaCas9 and an SaCas9 sgRNA corresponding to Sa sites 1–6 (SaR1–SaR6) were transfected in the same well. Dots represent individual biological replicates, and bar values represent mean±s.d. from n=3 independent biological replicates. Source data are provided as a Source Data file.

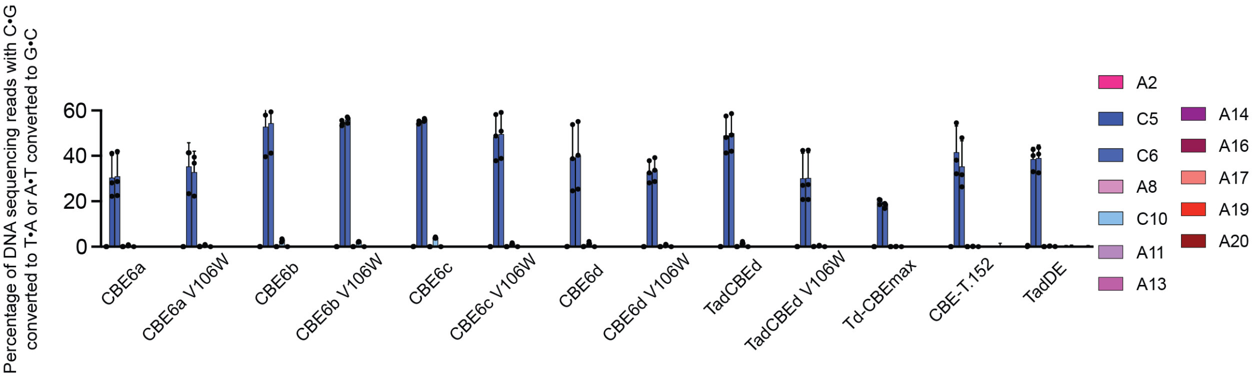

Supplementary Figure 22. On-target editing at *EMX1* correlated to RNA off-target editing. CBE6 variants along with existing cytosine base editors with SpCas9 nickases in the BE4max architecture were transfected into HEK293T cells in two plates. In one plate, RNA was harvested 48 hours after transfection, and in the other plate, genomic DNA was harvested. The genomic DNA was analyzed for on-target editing of *EMX1*. Dots represent individual values, and bar values represent mean±s.d. of n=3 independent biological replicates. Source data are provided as a Source Data file.

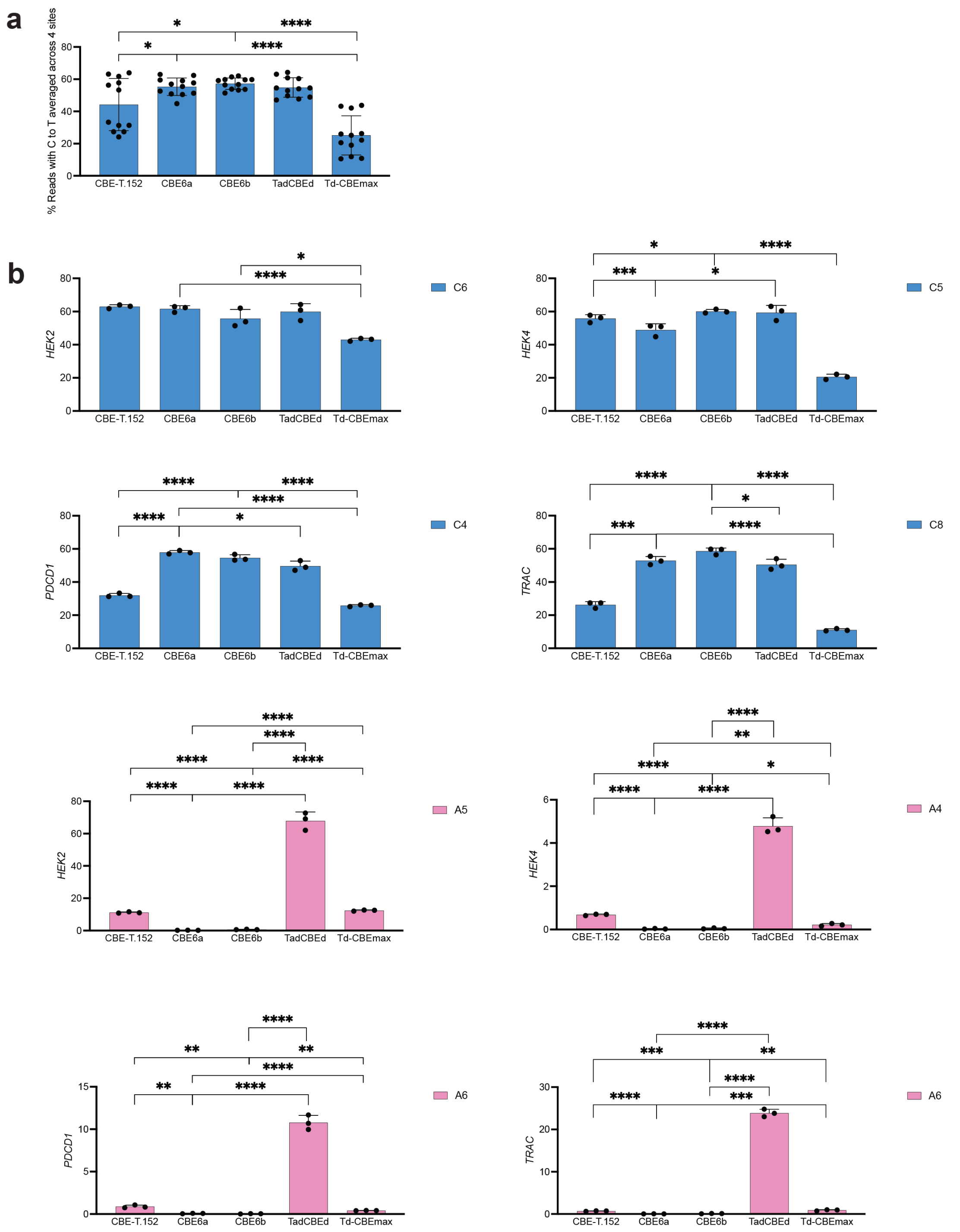

Supplementary Figure 23. Comparison of peak editing of base editors at four SpCas9 target sites with statistical significance. Each bar value is correlated to the peak editing presented in Supplementary Figure 12. *P* values were derived from a Student's two-tailed, unpaired *t*-test either from **(a)** an average of all sites or **(b)** per site tested. CBE6a and CBE6b were compared to TadCBEd, CBE-T.152, and Td-CBEmax individually. Dots represent individual values, and bar values represent mean±s.d. of n=3 independent biological replicates. HEK293T site 2 is abbreviated *HEK2*, and HEK293T site 4 is abbreviated *HEK4*. Source data are provided as a Source Data file.

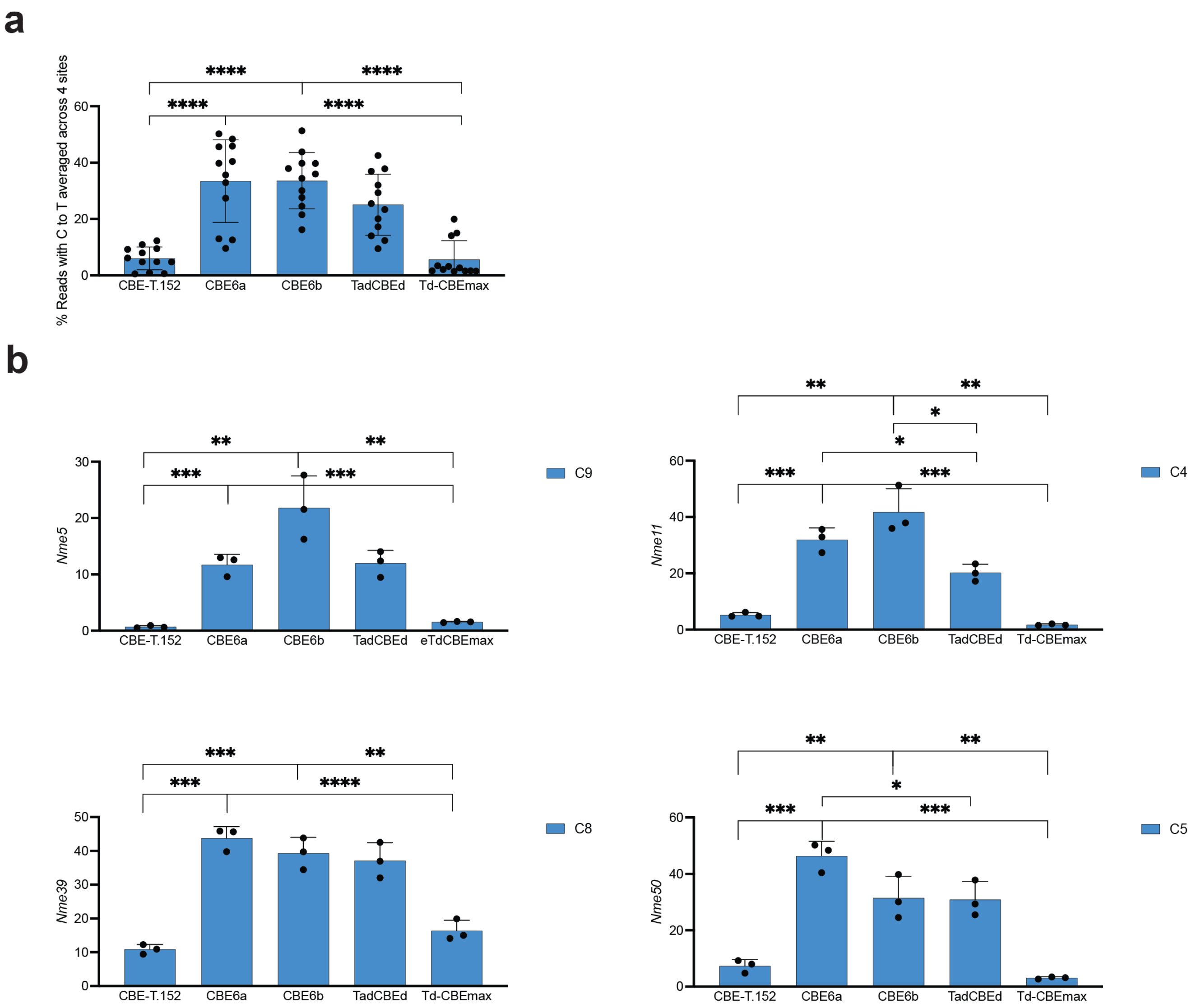

Supplementary Figure 24. Comparison of peak editing of base editors at four eNme2C-Cas9 target sites with statistical significance. Each bar value is correlated to the peak editing presented in Supplementary Figure 13. *P* values were derived from a Student's two-tailed, unpaired *t*-test either from **(a)** an average of all sites or **(b)** per site tested. CBE6a and CBE6b were compared to TadCBEd, CBE-T.152, and Td-CBEmax individually. Dots represent individual values, and bar values represent mean±s.d. of n=3 independent biological replicates. Source data are provided as a Source Data file.

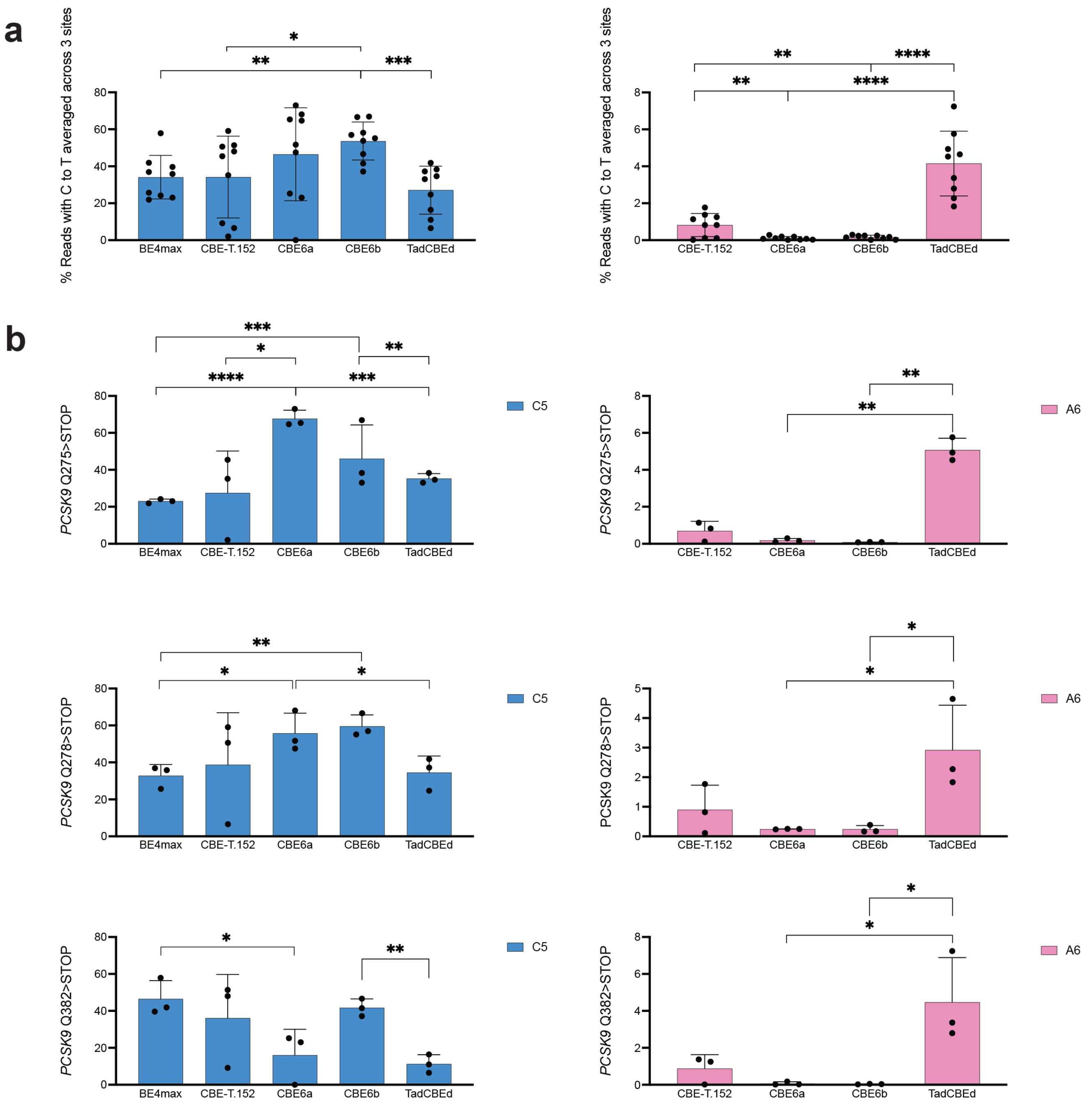

Supplementary Figure 25. Peak editing of base editors at 3 *PCSK9* target sites with statistical significance. Each bar value is correlated to the peak editing presented in Figure 5. *P* values were derived from a Student's two-tailed, unpaired *t*-test either from **(a)** an average of all sites or **(b)** per site tested. CBE6a and CBE6b were compared to TadCBEd, CBE-T.152, and BE4max individually. Dots represent individual values, and bar values represent mean±s.d. of n=3 independent biological replicates. Source data are provided as a Source Data file.

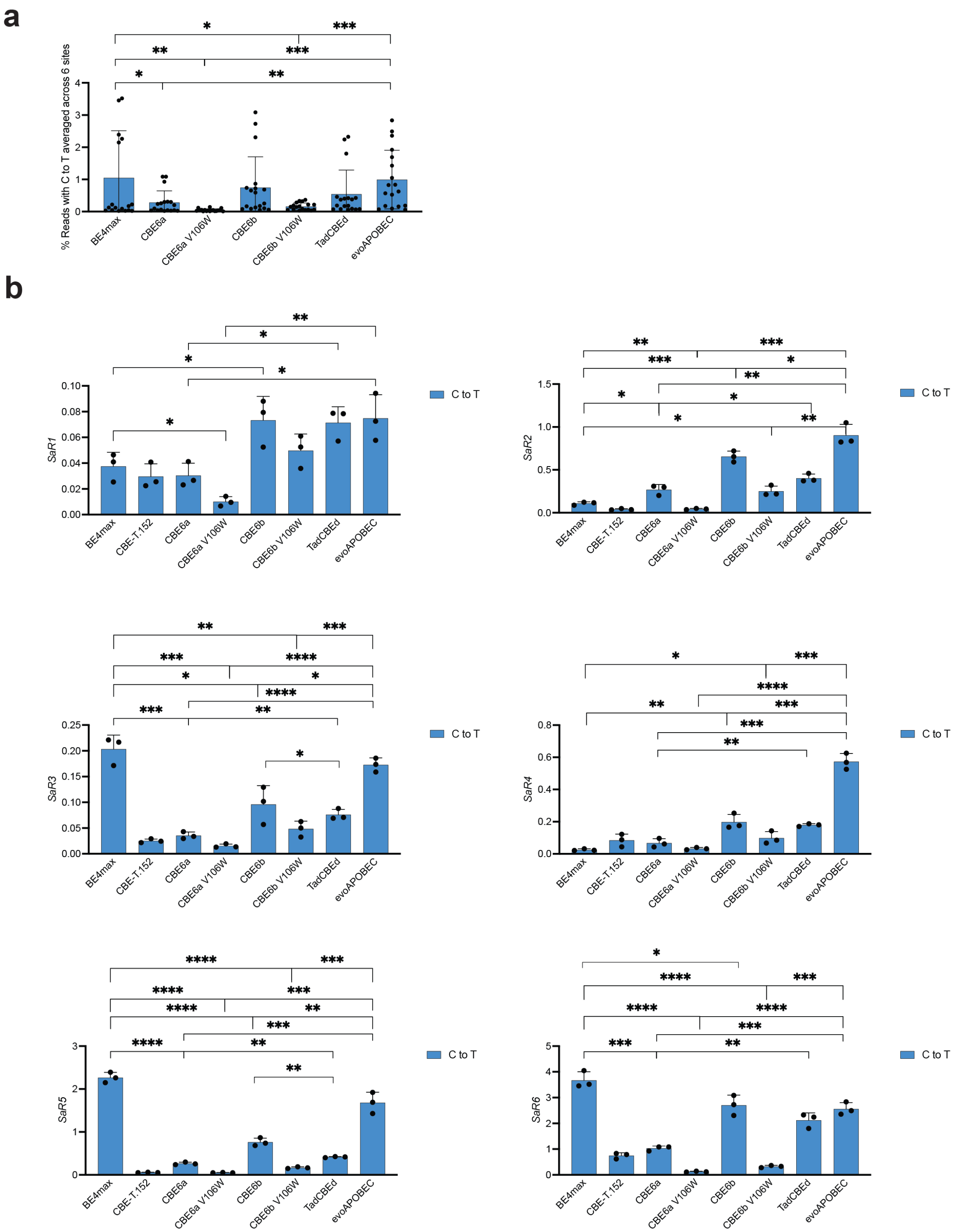

Supplementary Figure 26. Cas-independent off-target editing at six genomic SaCas9 R-loops with statistical significance. Each bar value is correlated to the peak editing presented in Figure 4a. *P* values were derived from a Student's two-tailed, unpaired *t*-test either from **(a)** an average of all sites or **(b)** per site tested. CBE6a and CBE6b were compared to TadCBEd, evoAPOBEC and BE4max individually. CBE6a V106W and CBE6b V106W were compared to evoAPOBEC and BE4max individually. Dots represent individual values, and bar values represent mean±s.d. of n=3 independent biological replicates. Source data are provided as a Source Data file.

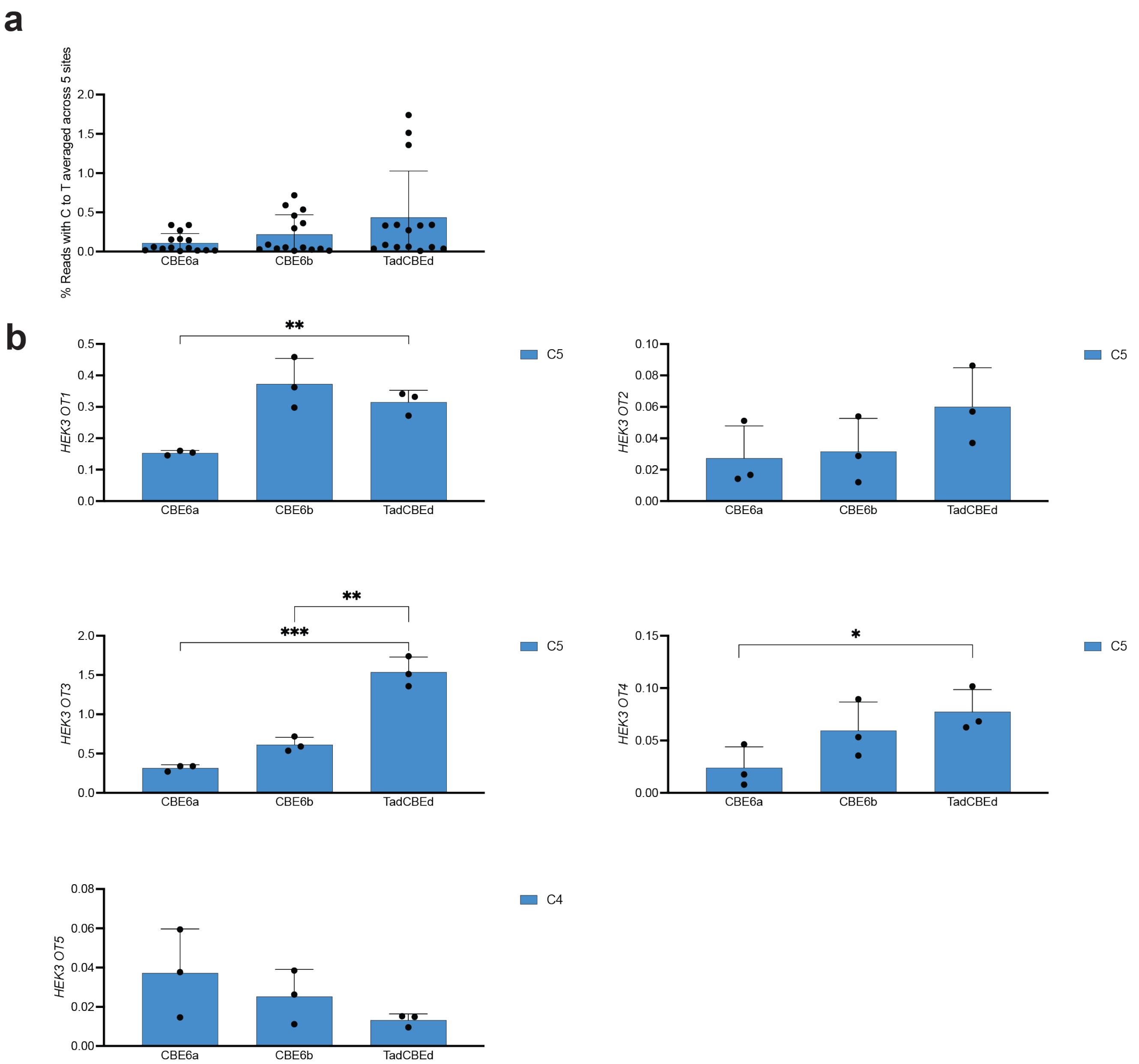

Supplementary Figure 27. Cas-dependent off-target editing of peak editing of known off-target sites for *HEK3* with statistical significance. Each bar value is correlated to the peak editing presented in Supplementary Figure 16. *P* values were derived from a Student's two-tailed, unpaired *t*-test either from **(a)** an average of all sites or **(b)** per site tested. CBE6a and CBE6b were compared to TadCBEd. Dots represent individual values, and bar values represent mean±s.d. of n=3 independent biological replicates. HEK293T site 3 is abbreviated *HEK3*. Source data are provided as a Source Data file.

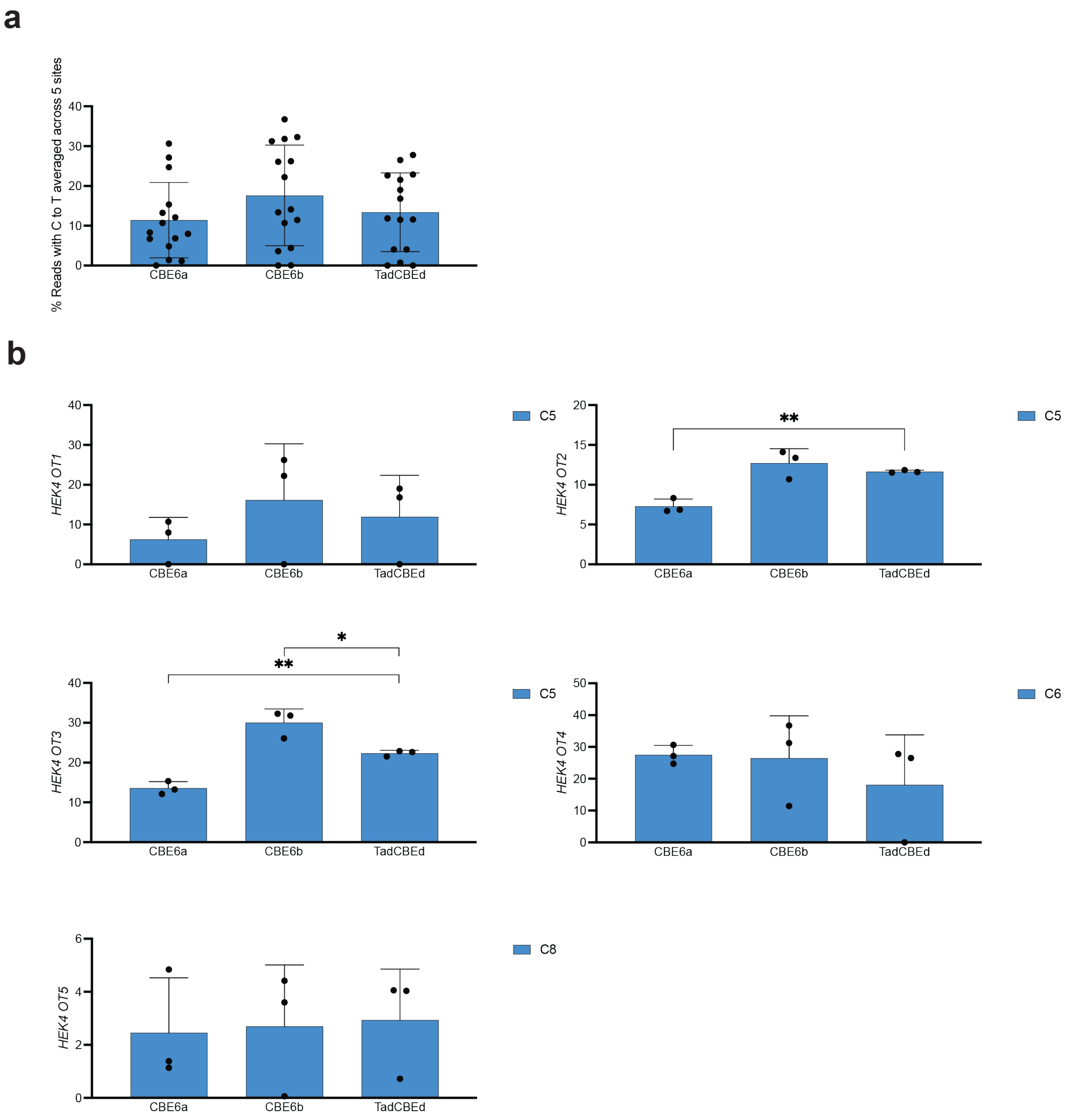

Supplementary Figure 28. Cas-dependent off-target editing of peak editing of known off-target sites for *HEK4* with statistical significance. Each bar value is correlated to the peak editing presented in Supplementary Figure 17. *P* values were derived from a Student's two-tailed, unpaired *t*-test either from **(a)** an average of all sites or **(b)** per site tested. CBE6a and CBE6b were compared to TadCBEd. Dots represent individual values, and bar values represent mean±s.d. of n=3 independent biological replicates. HEK293T site 4 is abbreviated *HEK4*. Source data are provided as a Source Data file.

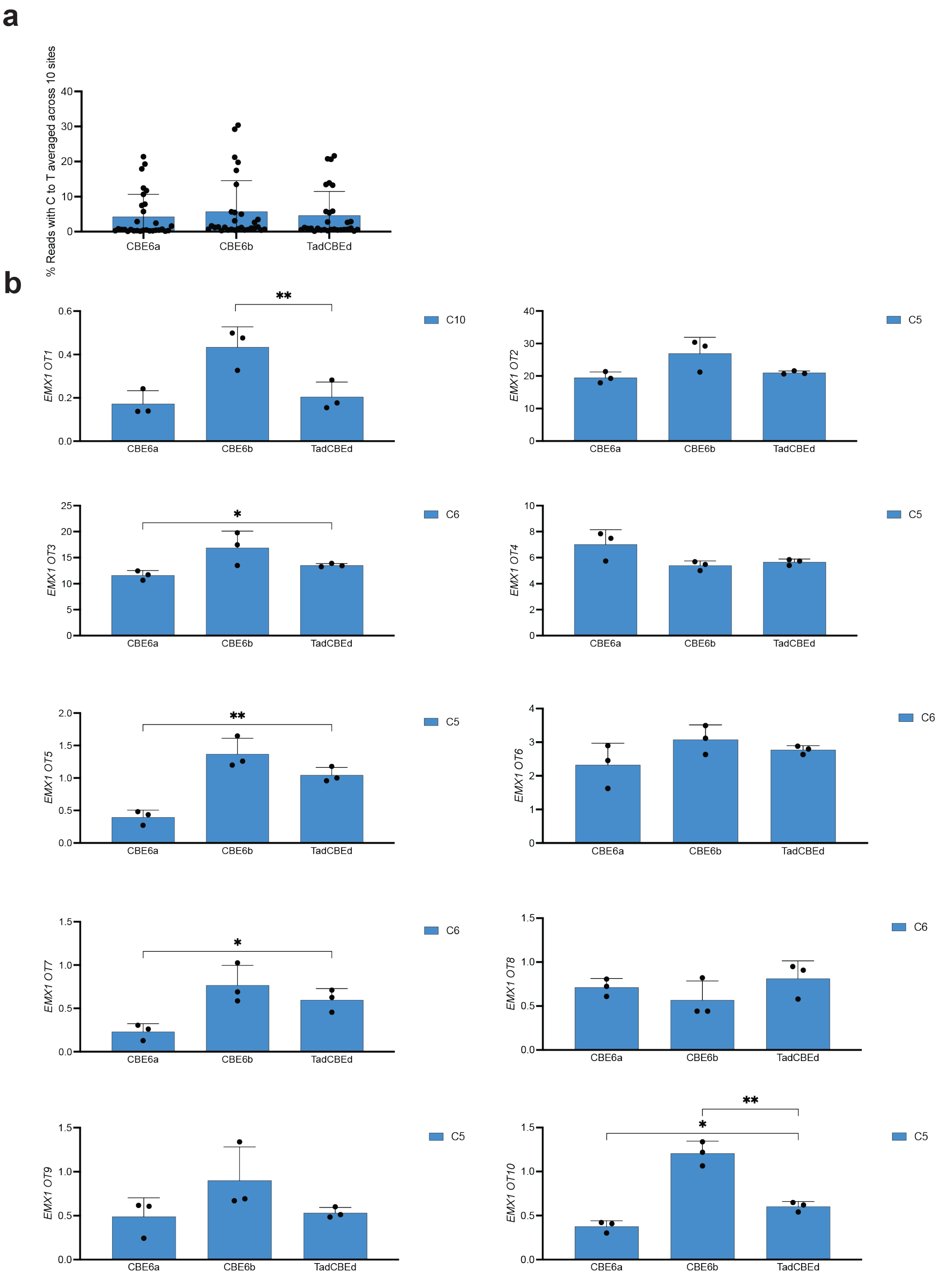

Supplementary Figure 29. Cas-dependent off-target editing of peak editing of known off-target sites for *EMX1* with statistical significance. Each bar value is correlated to the peak editing presented in Supplementary Figure 18. *P* values were derived from a Student's two-tailed, unpaired *t*-test either from **(a)** an average of all sites or **(b)** per site tested. CBE6a and CBE6b were compared to TadCBEd. Dots represent individual values, and bar values represent mean±s.d. of n=3 independent biological replicates. Source data are provided as a Source Data file.

a

b

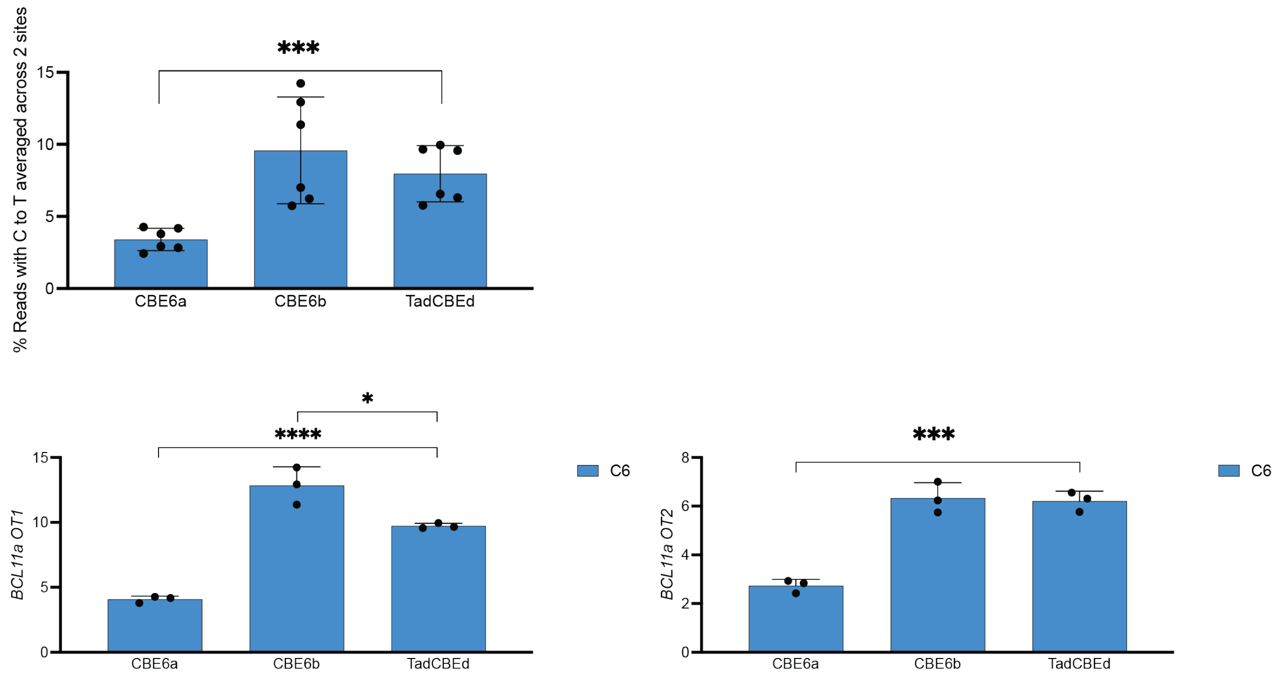

Supplementary Figure 30. Cas-dependent off-target editing of peak editing of known off-target sites for *BCL11a* with statistical significance. Each bar value is correlated to the peak editing presented in Supplementary Figure 19. *P* values were derived from a Student's two-tailed, unpaired *t*-test either from **(a)** an average of all sites or **(b)** per site tested. CBE6a and CBE6b were compared to TadCBEd. Dots represent individual values, and bar values represent mean±s.d. of n=3 independent biological replicates. Source data are provided as a Source Data file.

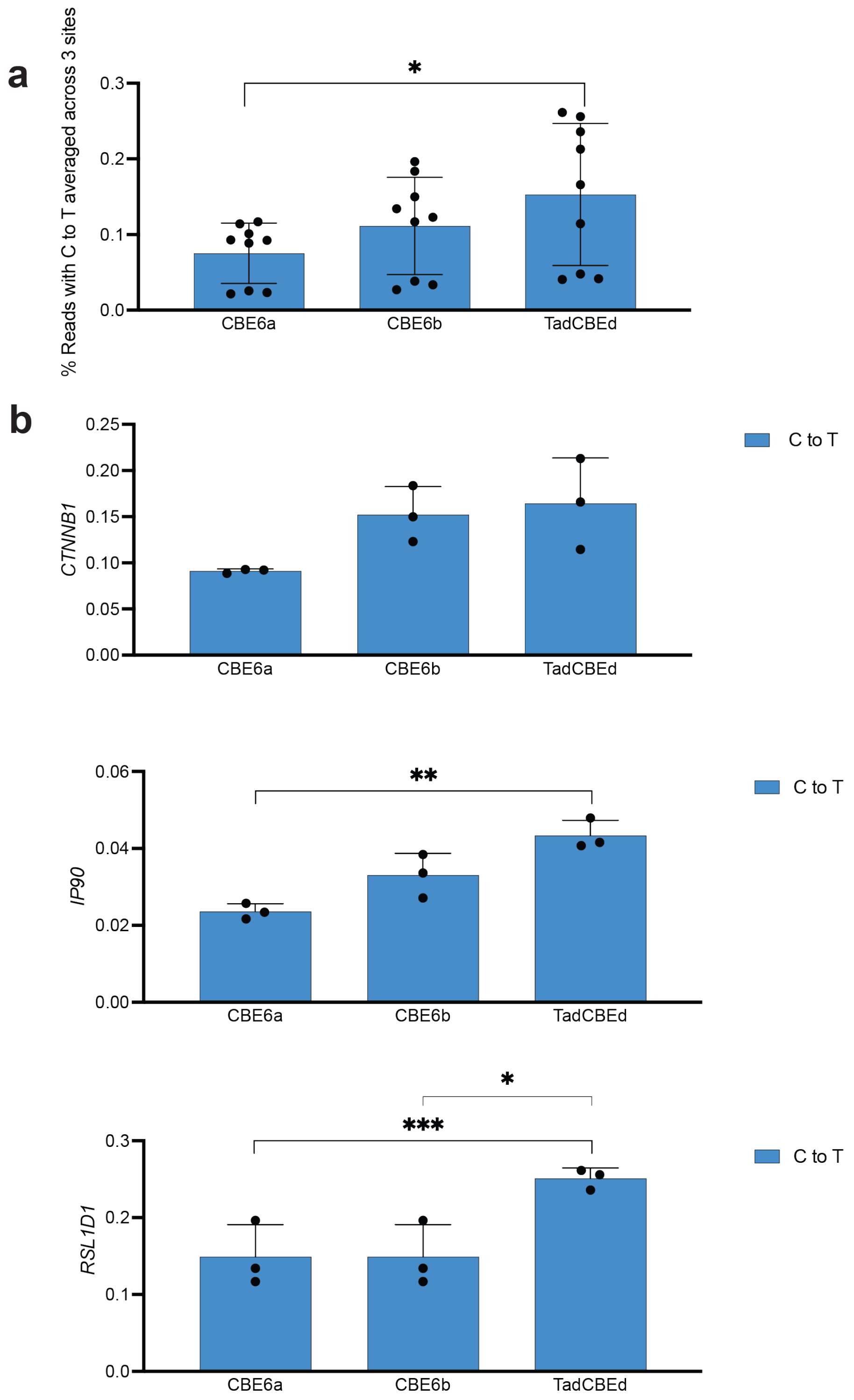

Supplementary Figure 31. RNA off-target editing with statistical significance.
Each bar value is correlated to the peak editing presented in Figure 4b. *P* values were derived from a Student's two-tailed, unpaired *t*-test either from **(a)** an average of all sites or **(b)** per site tested. CBE6a and CBE6b were compared to TadCBEd. Dots represent individual values, and bar values represent mean±s.d. of n=3 independent biological replicates. Source data are provided as a Source Data file.

Supplementary Table 1. Selectivity of TadDE-evolved CBEs calculated from *E. coli* profiling library (SD8, position 6). Selectivity is defined as the ratio of (mean CBE editing at position 6) to (mean ABE editing at position 6).

Variant	Selectivity
TadCBEd	10.6
CBE6a	990.9
CBE6b	2089.0
CBE6c	1146.4
CBE6d	1298.7
CBE-T1.52	185.6
Td-CBEmax	224.6

Supplementary Table 2. Selectivity of TadDE-evolved CBEs calculated from *E. coli* profiling library (sd2, positions 4-8). Selectivity is defined as the geometric mean of (the ratio of (mean CBE editing at each position) to (mean ABE editing at each position)).

Variant	Selectivity
TadCBEd	15.79
CBE6a	43.99
CBE6b	57.27
CBE6c	26.97
CBE6d	86.09
CBE-T1.52	53.23
Td-CBEmax	4.137

Supplementary Table 3. Plasmids and selection phage (SP) used in this work.

Name	Usage (resistance)	Origin	ORF1	ORF2
pEZ0056	Mammalian expression, SpCas9	pUC	pCMV.ABE8e-UGI-UGI	
pEZ0058	Mammalian expression, SpCas9	pUC	pCMV.TadCBEd	
pEZ0062	Mammalian expression, SpCas9	pUC	pCMV.CBE-T1.52	
pEZ0064	Mammalian expression, SpCas9	pUC	pCMV.eTdCBEmax	
pEZ0069	Mammalian expression, eNme2-C S6P	pUC	pCMV.CBE-T.152 (eNme2-C S6P)	
pEZ0071	Mammalian expression, eNme2-C S6P	pUC	pCMV.eTdCBEmax	
pEZ0116	Mammalian expression, SpCas9	pUC	pCMV.TadDE N46V Y73P	
pEZ0119	Mammalian expression, SpCas9	pUC	pCMV.TadDEN46L Y73P	
pEZ0120	Mammalian expression, SpCas9	pUC	pCMV.TadDE N46C Y73P	
pEZ0122	Mammalian expression, eNme2-C S6P	pUC	pCMV.TadDE N46V Y73P (eNme2-C S6P)	
pEZ0125	Mammalian expression, eNme2-C S6P	pUC	pCMV.TadDE N46L Y73P (eNme2-C S6P)	
pEZ0126	Mammalian expression, eNme2-C S6P	pUC	pCMV.TadDE N46C Y73P (eNme2-C S6P)	
pEZ0138	Mammalian expression, SpCas9	pUC	pCMV.TadDE N46V Y73P V106W	
pEZ0139	Mammalian expression, SpCas9	pUC	pCMV.TadDE N46L Y73P V106W	
pEZ0140	Mammalian expression, SpCas9	pUC	pCMV.TadDE N46C Y73P V106W	
pEZ0141	Mammalian expression, SpCas9	pUC	pCMV.TadDE N46I Y73P V106W	
pEZ0149	TadDE N46V Y73P IVT template	pUC		
pEZ0150	TadDE N46L Y73P IVT template	pUC		
pEZ0151	TadDE N46C Y73P IVT template	pUC		
pEZ0152	TadDE N46I Y73P IVT template	pUC		
pEZ0157	E. coli Library TadCBEd N46V (SpR)	SC101	pBAD.SD8.TadCBEd N46V.dSpCas9-UGI-UGI	
pEZ0158	E. coli Library TadCBEd N46L (SpR)	SC101	pBAD.SD8.TadCBEd N46L.dSpCas9-UGI-UGI	
pEZ0159	E. coli Library TadCBEd N46C (SpR)	SC101	pBAD.SD8.TadCBEd N46C.dSpCas9-UGI-UGI	
pEZ0160	E. coli Library TadCBEd N46I (SpR)	SC101	pBAD.SD8.TadCBEd N46I.dSpCas9-UGI-UGI	
pEZ0165	E. coli Library TadDE N46V Y73S (SpR)	SC101	pBAD.SD8.TadDE N46V.dSpCas9-UGI-UGI	
pEZ0166	E. coli Library TadDE N46L Y73S (SpR)	SC101	pBAD.SD8.TadDE N46L.dSpCas9-UGI-UGI	
pEZ0167	E. coli Library TadDE N46C Y73S (SpR)	SC101	pBAD.SD8.TadDE N46C.dSpCas9-UGI-UGI	
pEZ0168	E. coli Library TadDE N46I Y73S (SpR)	SC101	pBAD.SD8.TadDE N46I.dSpCas9-UGI-UGI	
pEZ0169	E. coli Library TadCBEd N46V Y73P (SpR)	SC101	pBAD.SD8.TadDE N46V Y73P.dSpCas9-UGI-UGI	
pEZ0170	E. coli Library TadCBEd N46L Y73P (SpR)	SC101	pBAD.SD8.TadDE N46L Y73P.dSpCas9-UGI-UGI	
pEZ0171	E. coli Library TadCBEd N46C Y73P (SpR)	SC101	pBAD.SD8.TadDE N46C Y73P.dSpCas9-UGI-UGI	
pEZ0172	E. coli Library TadCBEd N46I Y73P (SpR)	SC101	pBAD.SD8.TadDE N46I Y73P.dSpCas9-UGI-UGI	
pEZ0177	CBE-T1.52 IVT template	pUC		
SPEZ0001	PA(N)CE, ΔgIII SP	M13 f1	p_gIII.sD4.TadDE-NpuN	
SPEZ0065	PA(N)CE, ΔgIII SP	M13 f1	p_gIII.sD4.NNK at N46 on TadDE-NpuN	
pMN573	APOBEC1 (BE4max) IVT template	pUC		
pMN574	evoFERNY IVT template	pUC		
pMN575	evoAPOBEC IVT template	pUC		
pMN580	TadCBEd IVT template	pUC		
pMN582	YE1 IVT template	pUC		
pMN599	Mammalian expression, eNme2-C S6P	pUC	pCMV.ABE8e-UGI-UGI (eNme2-C S6P)	
pMN605	Mammalian expression, eNme2-C S6P	pUC	pCMV.TadCBEd (eNme2-C S6P)	
pMN607	Mammalian expression, eNme2-C S6P	pUC	pCMV.APOBEC1 (eNme2-C S6P)	
pMN608	Mammalian expression, eNme2-C S6P	pUC	pCMV.evoFERNY (eNme2-C S6P)	
pMN609	Mammalian expression, eNme2-C S6P	pUC	pCMV.evoAPOBEC (eNme2-C S6P)	
pMN790	Mammalian expression, eNme2-C S6P	pUC	pCMV.TadDE N46I Y73P (eNme2-C S6P)	
pMN784	Mammalian expression, SpCas9	pUC	pCMV.TadDE N46I Y73P	
pMN793	E. coli Library TadCBEd (SpR)	SC101	pBAD.sd2.TadCBEd.dSpCas9-UGI-UGI	
pMN794	E. coli Library ABE8e (SpR)	SC101	pBAD.sd2.ABE8e.dSpCas9-UGI-UGI	
pMN795	E. coli Library TadDE N46I Y73P (SpR)	SC101	pBAD.sd2.TadDE N46I Y73P.dSpCas9-UGI-UGI	
pMN816	E. coli Library ABE8e (SpR)	SC101	pBAD.SD8.ABE8e.dSpCas9-UGI-UGI	
pMN817	E. coli Library TadCBEd (SpR)	SC101	pBAD.SD8.TadCBEd.dSpCas9-UGI-UGI	
pMN818	E. coli Library TadDE (SpR)	SC101	pBAD.SD8.TadDE.dSpCas9-UGI-UGI	
pMN819	E. coli Library TadDE N46I Y73P (SpR)	SC101	pBAD.SD8.TadDE N46I Y73P.dSpCas9-UGI-UGI	
pMN831	E. coli Library CBE-T1.52 (SpR)	SC101	pBAD.SD8.CBE-T1.52.dSpCas9-UGI-UGI	
pMN832	E. coli Library eTdCBEmax (SpR)	SC101	pBAD.SD8.eTdCBEmax.dSpCas9-UGI-UGI	
pMN833	E. coli Library TadDE N46C Y73P (SpR)	SC101	pBAD.SD8.TadDE N46C Y73P.dSpCas9-UGI-UGI	
pMN834	E. coli Library TadDE N46L Y73P (SpR)	SC101	pBAD.SD8.TadDE N46L Y73P.dSpCas9-UGI-UGI	
pMN835	E. coli Library TadDE N46V Y73P(SpR)	SC101	pBAD.SD8.TadDE N46V Y73P.dSpCas9-UGI-UGI	
pMN837	E. coli Library TadDE N46C Y73P (SpR)	SC101	pBAD.sd2.TadDE N46C Y73P.dSpCas9-UGI-UGI	
pMN838	E. coli Library TadDE N46L Y73P (SpR)	SC101	pBAD.sd2.TadDE N46L Y73P.dSpCas9-UGI-UGI	
pMN839	E. coli Library TadDE N46V Y73P (SpR)	SC101	pBAD.sd2.TadDE N46V Y73P.dSpCas9-UGI-UGI	
pMN841	E. coli Library CBE-T1.52 (SpR)	SC101	pBAD.sd2.CBE-T1.52.dSpCas9-UGI-UGI	
pMN842	E. coli Library eTdCBEmax (SpR)	SC101	pBAD.sd2.eTdCBEmax.dSpCas9-UGI-UGI	
pBT44c	Circuit (KmR)	p15a	proC.SD8.intC.dCas9.UGI	
pBT120b	Circuit (CbR)	SC101-E93K	proT7.sd8.gIII.luxAB	proLac.sgRNA
pBT2dR3-proA	Circuit (SpR)	ColE1	proA.SynRBS 0.4k.T7-RNAP-degron	
pBT2dR3-proB	Circuit (SpR)	ColE1	proB.SynRBS 0.4k.T7-RNAP-degron	
pBT2dR3-proC	Circuit (SpR)	ColE1	proC.SynRBS 0.4k.T7-RNAP-degron	
pBT2dR3-proD	Circuit (SpR)	ColE1	proD.SynRBS 0.4k.T7-RNAP-degron	
pBT280	Mammalian expression, SpCas9	pUC	pCMV.evoFERNY	
pBT281	Mammalian expression, SpCas9	pUC	pCMV.evoAPOBEC	
pBT290	Mammalian expression, SpCas9	pUC	pCMV.APOBEC1 (BE4max)	

Supplementary Table 4. Promoter and RBS sequences for plasmids and phage used in evolution.

Name	Promoter/RBS	Description	Sequence
SP	PgIII	Promoter for TadA*-NpuN on phage	AATTCACCTCGAAAGCAAGTTGATAAAC<br>TGATACAATTAAAGGCTCCT
SP	RBS	RBS for expression of TadA*-NpuN on phage	AAGGAGGAAAA
P1	ProC	Promoter for NpuC-dCas9-ugi	CACAGCTAACACCACGTCGTCCCTATCT<br>GCTGCCCTAGGTCTATGAGTGGTTGCTG<br>GATAACTTTACGGGCATGCATAAGGCTC<br>GTATGATATATTCAGGGAGACCACAACG<br>GTTTCCCTCTACAAATAATTTTGTTTAACT<br>TTTACTAGAGTGGGACCCTACCTGCAGG<br>TGCAGT
P1	SD8	RBS for expression of NpuC-dCas9-ugi	AAGGAGGAAAAAAAA
P2	pLac	Promoter for sgRNA	GGCTTTACACTTTATGCTTCCGGCTCGTA<br>TGTTGTGTGG
P2	proT7	T7 promoter controls gIII transcription	TAATACGACTCACTATAGGGAGA
P2	sd8	RBS for expression of gIII	AAGGAAAAAAAAA
P3	ProA	Promoter for T7RNAP. Varies by stringency (A is weakest, most stringent)	CACAGCTAACACCACGTCGTCCCTATCT<br>GCTGCCCTAGGTCTATGAGTGGTTGCTG<br>GATAACTTTACGGGCATGCATAAGGCTC<br>GTAGGCTATATTCAGGGAGACCACAACG<br>GTTTCCCTCTACAAATAATTTTGTTTAACT<br>TTTACTAGAGTGGGACCCTACCTGCAGG<br>TGCAGT
P3	ProB	Promoter for T7RNAP. Varies by stringency.	CACAGCTAACACCACGTCGTCCCTATCT<br>GCTGCCCTAGGTCTATGAGTGGTTGCTG<br>GATAACTTTACGGGCATGCATAAGGCTC<br>GTAATATATATTCAGGGAGACCACAACG<br>GTTTCCCTCTACAAATAATTTTGTTTAACT<br>TTTACTAGAGTGGGACCCTACCTGCAGG<br>TGCAGT
P3	ProC	Promoter for T7RNAP. Varies by stringency.	CACAGCTAACACCACGTCGTCCCTATCT<br>GCTGCCCTAGGTCTATGAGTGGTTGCTG<br>GATAACTTTACGGGCATGCATAAGGCTC<br>GTATGATATATTCAGGGAGACCACAACG<br>GTTTCCCTCTACAAATAATTTTGTTTAACT<br>TTTACTAGAGTGGGACCCTACCTGCAGG<br>TGCAGT
P3	ProD	Promoter for T7RNAP. Varies by stringency (D is strongest, least stringent)	CACAGCTAACACCACGTCGTCCCTATCT<br>GCTGCCCTAGGTCTATGAGTGGTTGCTG<br>GATAACTTTACGGGCATGCATAAGGCTC<br>GTATAATATATTCAGGGAGACCACAACG<br>GTTTCCCTCTACAAATAATTTTGTTTAACT<br>TTTACTAGAGTGGGACCCTACCTGCAGG<br>TGCAGT
P3	RBSR3	RBS for expression of T7 RNAP	ACTACATCATCAGGC
Library	sd2	RBS for expression of the editor	AAAAAAAAAGGAAA
Library	SD8	RBS for expression of the editor	AAGGAGGAAAAAAAA
Library	sd5	RBS for expression of the editor	AAAAAAGGAAAAAA

Supplementary Table 5. cDNA amplicon sequences and primers for RNA off-target analysis.

Name	Amplicon	HTS-F	HTS-R
RSL1D1	TTGGCTTTCCAAATCAGTGGGTCTGACTTGAGGTCTGTGATG TGACCCTTTTCCTCACCTGCTCAACCATTATTCAC ATGGACTCCATCATATTCATTTGTAGTCATTCCCAGAGT GGCCCAGTGAGGGTCTCGCTGTATGAGAGTCGGCTAC GGAATTTAGGAGAAACAGAAGTTTCTTGGCTTTCATGCT GAGCTTGTTGGTCTAAGCTTATGAG	ACACTCTTTCCCT ACACGACGCTCT TCCGATCTNNNN TGGCTTTCCAAAT CAGTGGGTC	TGGAGTTCAGACG TGTGCTCTTCCGA TCTCTCATAAGCTT AGACCAACAAGC
CTNNB1	TTTGATGGAGTTGGACATGGCCATGGAACCAGACAGAAAAG CGGCTGTTAGTCACTGGCAGCAACAGTCTTACCTG GACTCTGGAATCCATTCTGGTGCCACTACCACAGCTCC TTCTCTGAGTGGTAAAGGCAATCCTGAGGAAGAGGATG TGGATACCTCCCAAGTCCTGTATGAGTGGGAACAGGGA TTTTCTCAGTCCTTCACTCAAGAACAAGTAGCTGG	ACACTCTTTCCCT ACACGACGCTCT TCCGATCTNNNN ATTTGATGGAGTT GGACATGGCC	TGGAGTTCAGACG TGTGCTCTCCAGC TACTTGTTCTTGAG TGAAGG
IP90	CTGGTTGACCAATCTGTGGTGAATAGTGGAAATCTGCT CAATGACATGACTCCTCCTGTAAATCCTTCACGTGAAAT TGAGGACCCAGAAGACCGGAAGCCCGAGGATTGGGAT GAAAGACCAAAAATCCCAGATCCAGAAGCTGTCAAGCC AGATGACTGGGATGAAGATGCCCCTGCTAAGATTCCAG ATGAAGAGGCCACAAAACCCGAAGGCTGGTTAGATGAT GAGCCTGAGTACGTAC	ACACTCTTTCCCT ACACGACGCTCT TCCGATCTNNNN CTGGTTGACCAA TCTGTGGTG	TGGAGTTCAGACG TGTGCTCTCTGCG TCTGGATCAGGTA CG

Supplementary Table 6. Primers for generating base editor amplicons for IVT.

Name	Sequence
IVT-F	TCGAGCTCGGTACCTAATACGACTCACTATAAGG
IVT-R	TTTTTTTTTTTTTTTTTTTTTTTTTTTTTTTTTTTTTTTTTTTTTTTTTTTTTTTTTTCTTCCTACTCAGGCTTTATTCAAAGACCA

Supplementary Table 7. Chemically synthesized guide RNAs used for fibroblast experiments.

Site Name	Protospacer	PAM	Amplicon	HTS-F	HTS-R
PCSK9 Q275>STOP	AAGCCAGCTGGTCCAGCCTG	TGG	CGCAGCAGCATTTCCACTGGCTTTTGACCAAACATCAGGCCACAAAGTTGATCCCCAAAATTAACCATCACTCTGTGCCTGTAAGGGAGGGGCTGGGAAAGGGGAGCAGGTCTCCCCAAGGGGTGACCTTGGCTTTGTTCCTCCCAGGCCTGGAGTTTATTCGGAAAAGCCAGCTGGTCCAGCCTGTGGGGCCACTGGTGGTGCTGCTGCCCCTGGCGGGTGGGTACAGCCGCGTCCTCAACGCCGCCTGCCAGCGCCTGGCGAGGGCTGGGGTCGTGCTGGTCACC	ACACTCTTTCCCTACACGACGCTCTTCCGATCTNNNNCGCAGCAGCATTTCCACTGG	TGGAGTTCAGACGTGTGCTCTTCCGATCTGGTGACCAGCACGACCCCAG
PCSK9 Q278>STOP	GGTCCAGCCTGTGGGGCCAC	TGG	CGCAGCAGCATTTCCACTGGCTTTTGACCAAACATCAGGCCACAAAGTTGATCCCCAAAATTAACCATCACTCTGTGCCTGTAAGGGAGGGGCTGGGAAAGGGGAGCAGGTCTCCCCAAGGGGTGACCTTGGCTTTGTTCCTCCCAGGCCTGGAGTTTATTCGGAAAAGCCAGCTGGTCCAGCCTGTGGGGCCACTGGTGGTGCTGCTGCCCCTGGCGGGTGGGTACAGCCGCGTCCTCAACGCCGCCTGCCAGCGCCTGGCGAGGGCTGGGGTCGTGCTGGTCACC	ACACTCTTTCCCTACACGACGCTCTTCCGATCTNNNNCGCAGCAGCATTTCCACTGG	TGGAGTTCAGACGTGTGCTCTTCCGATCTGGTGACCAGCACGACCCCAG
PCSK9 Q382>STOP	GTCACAGAGTGGGACATCAC	AGG	GTCATCACAGTTGGGGCCACCAATGCCCAAGACCAGCCGGTGACCCTGGGGACTTTGGGGACCAACTTTGGCCGCTGTGTGGACCTCTTTGCCCCAGGGGAGGACATCATTGGTGCCTCCAGCGACTGCAGCACCTGCTTTGTGTCACAGAGTGGGACATCACAGGCTGCTGCCCACGTGGCTGGTAAGTCACCACCCCACTGCCTCGGCCACCGTGATGCTAACAGCCCCTTTGGCAGTCAGGGTCTGTGCCGGGACCTCCAGTGCCAGGCTCTGTGCAGGGGGACCAGAGATG	ACACTCTTTCCCTACACGACGCTCTTCCGATCTNNNNGTCATCACAGTTGGGGCCAC	TGGAGTTCAGACGTGTGCTCTTCCGATCTCATCTCTGGTCCCCCTGCAC

Supplementary Note 1. Evolved deaminase amino acid sequences.

CBE6a:

MSEVEFSHEYWMRHALTLAKRARDEGEAPVGAVLVLNNRVIGEGWIRRIGLHDPTAHAEIM
ALRQGGLVMQNPRLIDATLYVTFEPCVMCAGAMINSRIGRVVFGVRNSKRGAAGSLMNVLN
YPGMNHRVEITEGILADECAALLCDFYRMPRQVFNAQKKAQSSIN

CBE6b:

MSEVEFSHEYWMRHALTLAKRARDEGEAPVGAVLVLNNRVIGEGWVRRIGLHDPTAHAEIM
ALRQGGLVMQNPRLIDATLYVTFEPCVMCAGAMINSRIGRVVFGVRNSKRGAAGSLMNVLN
YPGMNHRVEITEGILADECAALLCDFYRMPRQVFNAQKKAQSSIN

CBE6c:

MSEVEFSHEYWMRHALTLAKRARDEGEAPVGAVLVLNNRVIGEGWLRRIGLHDPTAHAEIM
ALRQGGLVMQNPRLIDATLYVTFEPCVMCAGAMINSRIGRVVFGVRNSKRGAAGSLMNVLN
YPGMNHRVEITEGILADECAALLCDFYRMPRQVFNAQKKAQSSIN

CBE6d:

MSEVEFSHEYWMRHALTLAKRARDEGEAPVGAVLVLNNRVIGEGWCRRIGLHDPTAHAEIM
ALRQGGLVMQNPRLIDATLYVTFEPCVMCAGAMINSRIGRVVFGVRNSKRGAAGSLMNVLN
YPGMNHRVEITEGILADECAALLCDFYRMPRQVFNAQKKAQSSIN

Supplementary Note 2. Sequences of Cas9 domains.

SpCas9 D10A:

DKKYSIGLAIGTNSVGWAVITDEYKVPSKKFKVLGNTDRHSIKKNLIGALLFDSGETAEARLKR
TARRRYTRRKNRICYLQEIFSNEMAKVDDSFFHRLEESFLVEEDKKHERHPIFGNIVDEVAYH
EKYPTIYHLRKKLVDSTDKADLRLIYLALAHMIKFRGHFLIEGDLNPDNSDVDKLFIQLVQTYN
QLFEENPINASGVDAKAILSARLSKSRRLENLIAQLPGEKKNGLFGNLIALSLGLTPNFKSNFD
LAEDAKLQLSKDTYDDDLDNLLAQIGDQYADLFLAAKNLSDAILLSDILRVNTEITKAPLSASMI
KRYDEHHQDLTLLKALVRQQLPEKYKEIFFDQSKNGYAGYIDGGASQEEFYKFIKPILEKMDG
TEELLVKLNREDLLRKQRTFDNGSIPHQIHLGELHAILRRQEDFYPFLKDNREKIEKILTFRIPY
YVGPLARGNSRFAWMTRKSEETITPWNFEEVVDKGASAQSFIERMTNFDKNLPNEKVLPKH
SLLYEYFTVYNELTKVKYVTEGMRKPAFLSGEQKKAIVDLLFKTNRKVTVKQLKEDYFKKIEC

FDSVEISGVEDRFNASLGTYHDLLKIIKDKDFLDNEENEDILEDIVLTLTLFEDREMIEERLKTY
AHLFDDKVMKQLKRRRYTGWGRSRKLINGIRDKQSGKTILDFLKSDGFANRNFMQLIHDDSL
TFKEDIQKAQVSGQGDSLHEHIANLAGSPAIKKGILQTVKVVDELVKVMGRHKPENIVIEMAR
ENQTTQKGQKNSRERMKRIEEGIKELGSQILKEHPVENTQLQNEKLYLYYLQNGRDMYVDQ
ELDINRLSDYDVDHIVPQSFLKDDSIDNKVLTRSDKNRGKSDNVPSEEVVKKMKNYWRQLLN
AKLITQRKFDNLTKAERGGLSELDKAGFIKRQLVETRQITKHVAQILDSRMNTKYDENDKLIRE
VKVITLKSKLVSDFRKDFQFYKVREINNYHHAHDAYLNAVVGTALIKKYPKLESEFVYGDYKV
YDVRKMIAKSEQEIGKATAKYFFYSNIMNFFKTEITLANGEIRKRPLIETNGETGEIVWDKGRD
FATVRKVLSMPQVNIVKKTEVQTGGFSKESILPKRNSDKLIARKKDWDPKKYGGFDSPTVAY
SVLVVAKVEKGKSKKLKSVKELLGITIMERSSFEKNPIDFLEAKGYKEVKKDLIIKLPYSLFELE
NGRKRMLASAGELQKGNELALPSKYVNFLYLASHYEKLKGSPEDNEQKQLFEQHKHYLDEII
EQISEFSKRVILADANLDKVLSAYNKHRDKPIREQAENIIHLFTLTNLGAPAFKYFDT
TIDRKRYTSTKEVLDATLIHQSITGLYETRIDLSQLGGD

eNme2-C Cas9 (S6P mutation relative to reported eNme2-C sequence):
AAFKPNPINYILGLAIGIASVGWAMVEIDEEENPIRLIDLGVRVFERAEVPKTGDSLAMARRLA
RSVRRLTRRRAHRLLRARRLLKREGVLQAADFDENGLIKSLPNTPWQLRAAALDRKLTPLE
WSAVLLHLIKHRGYLSQRKNEGETADKELGALLKGVANNAHALQTGDFRTPAELALNKFEKE
SGHIRNQRGDYSHTFSRKDLQAELILLFEKQKEFGNPHVSGGLKEGIETLLMTQRPALSGDA
VQKMLGHCTFEPAEPKAAKNTYTAERFIWLTKLNNLRILEQGSERPTDTERATLMDEPYRKS
KLTYAQARKLLGLEDTAFFKGLRYGKDNAEASTLMEMKAYHAISRALEKEGLKDKKSPLNLS
SELQDEIGTAFSLFKTDEDITGRLKDRVQPEILEALLKHISFDKFVQISLKALRRIVPLMEQGKR
YDEACAEIYGDHYGKKNTEEKIYLPPIPADEIRNPVVLALSQARKVINGVVRRYGSPARIHIET
AREVGKSFKDRKEIEKRQEENRKDREKAAAKFRYFPNFVGEPKSKDILKLRLYEQQHGKCLY
SGKEINLVRLNEKGYVEIDAALPFSRTWDDFNNKVLVLGSENQNKGNQTPYEYFNGKDNSR
EWQEFKARVETSRFPRSKKQRILLQKFDEDGFKECNLNDTRYVNRFLCQFVADHILLTGKGK
RRVFASNGQITNLLRGFWGLRKVRAENDRHHALDAVVVACSTVAMQQKITRFVRYKEMNAF
DGKTIDKETGKVLHQKTHFPQPWEFFAQEVMIRVFGKPDGKPEFEEADTPEKLRTLLAEKLS
SRPEAVHEYVTPLFVRAPNRKMSGAHKDTLRSAKRFVKHNEKISVKRVWLTEIKLADLENMV
NYKNGREIELYELKARLEAYGGNAKQAFDPKDNPFYKKGGQLVKAVRVEKTQESGVLLNKK
NAYTIADNGDMVRVDVFCKVDKKGKNQYFIVPIYAWQVAENILPDIDCKGYRIDDSYTFCFSL
HKYDLIAFQKDEKSKVE
