# Supplementary material for: Phage-assisted evolution of highly active cytosine base editors with enhanced selectivity and minimal sequence context preference
Source: Nat Commun. 2024 Feb 24;15:1697. doi: 10.1038/s41467-024-45969-7 (PMC10894238; doi:10.1038/s41467-024-45969-7)
Supplement: Supplementary file 3 — Description of Additional Supplementary Files [file 41467_2024_45969_MOESM3_ESM.pdf]

Description of Additional Supplementary Files

Supplementary Data 1. Target protospacers and amplicons used in this study with corresponding primers for genomic amplification.

Supplementary Data 2. Barcodes for Library Analysis
