# Supplementary material for: Phage-assisted evolution of highly active cytosine base editors with enhanced selectivity and minimal sequence context preference
Source: Nat Commun. 2024 Feb 24;15:1697. doi: 10.1038/s41467-024-45969-7 (PMC10894238; doi:10.1038/s41467-024-45969-7)
Supplement: Supplementary file 6 — Reporting Summary [file 41467_2024_45969_MOESM6_ESM.pdf]

nature portfolio

Corresponding author(s): David R. Liu

Last updated by author(s): Dec 18, 2023

Reporting Summary

Nature Portfolio wishes to improve the reproducibility of the work that we publish. This form provides structure for consistency and transparency in reporting. For further information on Nature Portfolio policies, see our Editorial Policies and the Editorial Policy Checklist.

Statistics

For all statistical analyses, confirm that the following items are present in the figure legend, table legend, main text, or Methods section.

n/a	Confirmed	
☐	☒	The exact sample size (*n*) for each experimental group/condition, given as a discrete number and unit of measurement
☐	☒	A statement on whether measurements were taken from distinct samples or whether the same sample was measured repeatedly
☐	☒	The statistical test(s) used AND whether they are one- or two-sided<br>*Only common tests should be described solely by name; describe more complex techniques in the Methods section.*
☒	☐	A description of all covariates tested
☐	☒	A description of any assumptions or corrections, such as tests of normality and adjustment for multiple comparisons
☐	☒	A full description of the statistical parameters including central tendency (e.g. means) or other basic estimates (e.g. regression coefficient) AND variation (e.g. standard deviation) or associated estimates of uncertainty (e.g. confidence intervals)
☐	☒	For null hypothesis testing, the test statistic (e.g. *F*, *t*, *r*) with confidence intervals, effect sizes, degrees of freedom and *P* value noted<br>*Give P values as exact values whenever suitable.*
☒	☐	For Bayesian analysis, information on the choice of priors and Markov chain Monte Carlo settings
☒	☐	For hierarchical and complex designs, identification of the appropriate level for tests and full reporting of outcomes
☒	☐	Estimates of effect sizes (e.g. Cohen's *d*, Pearson's *r*), indicating how they were calculated

Our web collection on statistics for biologists contains articles on many of the points above.

Software and code

Policy information about availability of computer code

Data collection	High-throughput sequencing data was collected using Illumina Miseq Control software (3.1) and instruments. CRISPResso files were imported into Microsoft Excel (version 16.77) for analyses.
Data analysis	High-throughput sequencing data were demultiplexed using the MiSeq Reporter (3.1, Illumina), and fastq files were analyzed using Crispresso2 (v2.0.34). Microsoft Excel<br>(version 16.77) was used for analyses. Prism 10 (v10.0.3, GraphPad) was used to generate dot plots and bar plots of these data. Mutato is available as a docker image at https://hub.docker.com/r/araguram/mutato. Further details and references are provided in the Methods. Library data processing and analysis were performed with Python 3.9. Library analysis code can be found on Github at https://github.com/MLE-zhang/BE_Lib, and sequence logos were generated using Logomaker. PyRosetta was used for energy modeling.

For manuscripts utilizing custom algorithms or software that are central to the research but not yet described in published literature, software must be made available to editors and reviewers. We strongly encourage code deposition in a community repository (e.g. GitHub). See the Nature Portfolio guidelines for submitting code & software for further information.

Data

Policy information about availability of data

All manuscripts must include a data availability statement. This statement should provide the following information, where applicable:

- Accession codes, unique identifiers, or web links for publicly available datasets
- A description of any restrictions on data availability
- For clinical datasets or third party data, please ensure that the statement adheres to our policy

High-throughput DNA sequencing FASTQ files are available from the NCBI SRA under BioProject PRJNA1028129. Amino acid sequences of deaminases in this study are provided in the Supplementary Information
as Supplementary Note 1. The published structure of ABE8e (PDB ID 6VPC) can be accessed here: https://www.rcsb.org/structure/6vpc. Other data are available in Source Data files.
Plasmids encoding CBE6s are available on Addgene.

Research involving human participants, their data, or biological material

Policy information about studies with human participants or human data. See also policy information about sex, gender (identity/presentation), and sexual orientation and race, ethnicity and racism.

Reporting on sex and gender	NA- samples for fibroblasts were purchased as de-identified samples from the Coriell Institute for Medical Research and are IRB exempt.
Reporting on race, ethnicity, or other socially relevant groupings	NA- samples for fibroblasts were purchased as de-identified samples from the Coriell Institute for Medical Research and are IRB exempt.
Population characteristics	NA- samples for fibroblasts were purchased as de-identified samples from the Coriell Institute for Medical Research and are IRB exempt.
Recruitment	NA- samples for fibroblasts were purchased as de-identified samples from the Coriell Institute for Medical Research and are IRB exempt.
Ethics oversight	NA- samples for fibroblasts were purchased as de-identified samples from the Coriell Institute for Medical Research and are IRB exempt.

Note that full information on the approval of the study protocol must also be provided in the manuscript.

Field-specific reporting

Please select the one below that is the best fit for your research. If you are not sure, read the appropriate sections before making your selection.

☒ Life sciences ☐ Behavioural & social sciences ☐ Ecological, evolutionary & environmental sciences

For a reference copy of the document with all sections, see nature.com/documents/nr-reporting-summary-flat.pdf

Life sciences study design

All studies must disclose on these points even when the disclosure is negative.

Sample size	Sample sizes were n=3 independent biological replicates, which has been previously reported to yield reproducible values for mammalian gene editing experiments (Chen*, Hussmann*, et al. Cell (2021)). For the library experiment, sequencing depth was chosen to obtain a minimum average of 2000 reads per designed library oligos.
Data exclusions	For analysis of individual amplicons, no data were excluded.
Replication	For individual amplicons, biological triplicate experiments were performed with distinct aliquots of cells at intervals ranging from days to weeks between experiments. Findings have been replicated, and the base editors developed in this work have been independently tested by other researchers in the lab. For library experiments, two biological replicates were performed and were shown to be consistent.
Randomization	All independent biological replicates were treated identically, so randomization was not relevant.
Blinding	Mammalian cells used in this study were treated under identical conditions, so blinding was not relevant.

Reporting for specific materials, systems and methods

We require information from authors about some types of materials, experimental systems and methods used in many studies. Here, indicate whether each material, system or method listed is relevant to your study. If you are not sure if a list item applies to your research, read the appropriate section before selecting a response.

Materials & experimental systems

n/a	Involved in the study
☒	☐ Antibodies
☐	☒ Eukaryotic cell lines
☒	☐ Palaeontology and archaeology
☒	☐ Animals and other organisms
☒	☐ Clinical data
☒	☐ Dual use research of concern
☒	☐ Plants

Methods

n/a	Involved in the study
☒	☐ ChIP-seq
☒	☐ Flow cytometry
☒	☐ MRI-based neuroimaging

Eukaryotic cell lines

Policy information about cell lines and Sex and Gender in Research

Cell line source(s)	HEK293T were obtained from ATCC (ATCC CRL-3216). De-identified fibroblasts were obtained from the Coriell Institute for Medical Research (GM03348).
Authentication	Cells were authenticated by the supplier using STR analysis.
Mycoplasma contamination	HEK293T cell lines tested negative for mycoplasma.
Commonly misidentified lines (See ICLAC register)	No commonly misidentified cell lines were used.

Plants

Seed stocks	*Report on the source of all seed stocks or other plant material used. If applicable, state the seed stock centre and catalogue number. If plant specimens were collected from the field, describe the collection location, date and sampling procedures.*
Novel plant genotypes	*Describe the methods by which all novel plant genotypes were produced. This includes those generated by transgenic approaches, gene editing, chemical/radiation-based mutagenesis and hybridization. For transgenic lines, describe the transformation method, the number of independent lines analyzed and the generation upon which experiments were performed. For gene-edited lines, describe the editor used, the endogenous sequence targeted for editing, the targeting guide RNA sequence (if applicable) and how the editor was applied.*
Authentication	*Describe any authentication procedures for each seed stock used or novel genotype generated. Describe any experiments used to assess the effect of a mutation and, where applicable, how potential secondary effects (e.g. second site T-DNA insertions, mosiacism, off-target gene editing) were examined.*
